# *HES* and *Mox* genes are expressed during early mesoderm formation in a mollusk with putative ancestral features

**DOI:** 10.1038/s41598-021-96711-y

**Published:** 2021-09-09

**Authors:** Attila Sachslehner, Elisabeth Zieger, Andrew Calcino, Andreas Wanninger

**Affiliations:** Department of Evolutionary Biology, Unit for Integrative Zoology, University of Vienna, Althanstrasse 14, 1090 Vienna, Austria

**Keywords:** Developmental biology, Evolution, Zoology

## Abstract

The mesoderm is considered the youngest of the three germ layers. Although its morphogenesis has been studied in some metazoans, the molecular components underlying this process remain obscure for numerous phyla including the highly diverse Mollusca. Here, expression of *Hairy and enhancer of split* (*HES*), *Mox*, and *myosin heavy chain* (*MHC*) was investigated in *Acanthochitona fascicularis*, a representative of Polyplacophora with putative ancestral molluscan features. While *AfaMHC* is expressed throughout myogenesis, *AfaMox1* is only expressed during early stages of mesodermal band formation and in the ventrolateral muscle, an autapomorphy of the polyplacophoran trochophore. Comparing our findings to previously published data across Metazoa reveals *Mox* expression in the mesoderm in numerous bilaterians including gastropods, polychaetes, and brachiopods. It is also involved in myogenesis in molluscs, annelids, tunicates, and craniates, suggesting a dual role of *Mox* in mesoderm and muscle formation in the last common bilaterian ancestor. *AfaHESC2* is expressed in the ectoderm of the polyplacophoran gastrula and later in the mesodermal bands and in putative neural tissue, whereas *AfaHESC7* is expressed in the trochoblasts of the gastrula and during foregut formation. This confirms the high developmental variability of HES gene expression and demonstrates that *Mox* and *HES* genes are pleiotropic.

## Introduction﻿

Germ layers form early in animal development and give rise to the various adult tissues and cell types. The most ancient germ layers, the ectoderm and endoderm, are established during gastrulation, while the third germ layer, the mesoderm, is argued to be the youngest and probably evolved in the bilaterian lineage^1–4^, but see^5,6^ for alternative view. The mesoderm is considered a key innovation, since numerous bilaterian organ systems such as muscles, bone, and connective tissue derive from this germ layer^7^. In a number of protostomes, the mesoderm is formed by cells that immigrate from the blastopore margin into the blastocoel. These sometimes form a pair of mesodermal bands as, for example, in animals that exhibit spiral cleavage (the Spiralia; e.g., Platyhelminthes, Annelida, and Mollusca^8–12^). In several other protostomes, as well as in deuterostomes, the mesoderm-forming cells typically detach from the archenteron wall^1,13,14^. While the mesodermal cell lineage has been investigated in a number of lophotrochozoan representatives including the flatworm *Hoploplana*^8^, the polychaete annelids *Podarke*, *Polygordius*, and *Scoloplos*^15^, the gastropods *Planorbis*^16^ and *Crepidula*^17^, and the polyplacophoran mollusk *Acanthochitona*^9^, the molecular mechanisms underlying mesoderm specification remain largely unclear^12^.

*Myosin heavy chain* (*MHC*), *Mox*, and *Hairy and enhancer of split* (*HES*) genes are known to be expressed in mesoderm and/or early muscle formation in several bilaterians, but functional genetic studies are lacking for almost all taxa except for a very limited number of model organisms^3,7,18–21^. In Mollusca, one of the most diverse, abundant, and widespread animal phyla, the molecular underpinnings of mesoderm specification remain only poorly studied. Information on the expression of these three key factors are virtually non-existent and functional data are absent altogether.

*MHC* or *myosin class II* is a member of the myosin superfamily. It is, together with *myosin class I*, often assumed to constitute the most ancient myosin class, having evolved at the bikont-unikont split^22^. The protein products of *MHC* build the myosin fibres of cnidarian, ctenophore, and bilaterian muscle cells^23–26^. In the annelid *Platynereis dumerilii*, *MHC* is expressed in both, striated and smooth muscles of the early nectochaete larva^20^. In *Drosophila melanogaster*, *MHC* is expressed in somatic and visceral muscles as well as in cardioblasts^18^, and in the cephalochordate *Branchiostoma belcheri*, *MHC* expression is found during somite formation and in the notochord^27,28^. In vertebrates, *MHC* is involved in the development of skeletal, cardiac, and smooth muscles^29^. In the non-bilaterian cnidarian *Nematostella*, *MHC* transcripts are present in the tentacle muscles and in retractor muscles of primary polyps^25^. They are also found in muscle progenitor cells in the tentacle root of the ctenophore *Pleurobrachia pileus*^26^.

*Mox* genes possess a conserved helix-turn-helix DNA-binding homeodomain^30^. Previous studies have suggested a sister group relationship to the homeotic gene *even-skipped* (*Evx*)^31^. In chordates, *Mox* expression was reported during formation and differentiation of the main mesodermal derivatives, the somites, that give rise to muscles, bones, and connective tissue^13,32^. Expression of the *Drosophila Mox* ortholog *buttonless* is restricted to dorsal median cells which play a crucial role in axon guidance. Importantly, *buttonless* expression was not detected in *Drosophila* muscle progenitor cells or muscle tissue^33^, suggesting a loss of *Mox* in myogenesis in this lineage.

*HES* genes are members of the basic helix-loop-helix superfamily and direct downstream targets of the Delta-Notch signalling pathway^34^. They possess an additional *HES*-specific hairy orange domain and a WRPW motif at the C-terminal end^19^. *HES* genes are involved in a variety of developmental processes such as mesoderm formation, maintaining stem cell potential, or partitioning of morphological territories (e.g., segmentation in annelids, arthropods, chordates, as well as budding in *Hydra*)^19,31,35–37^. *HES* genes in mollusks have so far only been studied in the gastropod *Crepidula fornicata*, where one *HES* gene was found to be expressed around the mouth as well as in neurosensory cells in the early larva, while the other one shows more dynamic expression domains in the lateral ectoderm around the mouth^38^.

In order to test whether *MHC*, *Mox*, and *HES* are expressed during mesoderm formation in mollusks, we investigated tempo-spatial expression of *MHC*, *Mox*, and *HES* genes in *Acanthochitona fascicularis*, a member of Polyplacophora that displays several morphological characteristics thought to be ancestral for one of the two major molluscan lineages, the Aculifera^39,40^. In addition, we provide a metazoan-wide comparative survey on the tempo-spatial expression domains of these genes. By plotting these data on current phylogenies and by applying a ground pattern reconstruction approach using parsimony, we discuss scenarios concerning the emergence and loss of involvement of these genes in mesoderm formation and myogenesis across major lineages of the metazoan tree of life.

## Material and methods

### Animals and fixation

Adult *Acanthochitona fascicularis* specimens were collected in the intertidal region between the Station Biologique de Roscoff and the Île Verte in Roscoff, France (48° 43′ 44.70″ N 3° 59′ 13.53″ W). Adults and all developmental stages were maintained in glass dishes with filtered seawater at 18–21 °C. Spontaneous spawning of mature males and females generally occurred 1 to 3 days after collection. Gametes were inseminated by adding drops of sperm to the eggs. Upon the first observation of 2-cell stages (~ 80 min after fertilization), the embryos were washed multiple times with filtered sea water to prevent polyspermy and bacterial or fungal infection.

The gastrula stage was reached at around 8 h post fertilization (hpf). Trochophore larvae hatched from 18 hpf onwards. At 48–60 hpf, larvae reached the metamorphic competent stage (referred to as “late trochophore larva” herein). Early juveniles that had completed metamorphosis appeared between 60 and 90 hpf.

In order to fix samples for RNA extraction, specimens were centrifuged, the seawater was removed, and liquid nitrogen was added. Specimens were stored at − 80 °C until RNA extraction. For in situ hybridization experiments, specimens were fixed for 1–2 h in 4% paraformaldehyde (PFA Sigma-Aldrich #158127; St. Louis, USA) in MOPS-EGTA (0.1 M MOPS Sigma-Aldrich #69947; 2 mM MgSO_4_ Thermo Fisher Scientific #52044; Waltham, USA; 1 mM EGTA, Sigma-Aldrich #E4378; 0.5 M NaCl, Roth #HN00.1; Karlsruhe, Germany) and washed twice or thrice in ice cold 100% methanol. Fixed specimens were stored at − 20 °C.

### RNA probe design

Total RNA extraction from pooled developmental stages spanning early cleavage stages to juveniles was performed using the Qiagen RNeasy mini kit 50 (#74104; Venlo, Netherlands). Reverse transcription into cDNA was performed with the Roche 1st strand cDNA synthesis kit for RT-PCR (Roche #11483188001; Rotkreuz, Switzerland). Specific primers for each gene of interest were designed manually and purchased from Microsynth AG (Zürich, Switzerland) (Supplementary Table 1). Reading frames and orientation of the transcriptomic templates were assessed with the ExPASy translate tool^41^ (https://web.expasy.org/translate/). Melting temperatures of designed primers were assessed with the Promega Oligo Calculator tool^42^ (https://at.promega.com/resources/tools/biomath/tm-calculator/; 500 nM primer concentration, 5× green or colourless GoTaq Reaction Buffer) and self-complementary check was done with the Northwestern biotools OligoCalc tool^43^ (http://biotools.nubic.northwestern.edu/OligoCalc.html). The genes of interest were amplified by PCR (Promega protocol #9PIM829; 5× Go-Taq Flexi Buffer Promega #M890A, Fitchburg, USA; magnesium chloride, Promega #A351; dNTP Mix, Promega #1141; Go Taq Flexi DNA Polymerase, Promega #M780B) and the gene-specific primers. The amplified genes were ligated into a pGEM-T easy vector (Promega #A1380). The plasmid was amplified using *E. coli* competent cells (Promega #L2001). Plasmid DNA was purified using the QIAprep spin miniprep kit 250 (Qiagen #27106). Inserts were sequenced by Microsynth AG (Vienna) using sp6 primers. Amplification of the insert was done by PCR (Promega protocol #9PIM829; M13 Primer, Microsynth AG). In vitro transcription was done using the DIG RNA Labeling Mix, 10× conc. (Roche #11277073910) with either T7 RNA polymerase (Roche #10881767001) or sp6 RNA polymerase (Roche #10810274001). Additionally, 1 µl of DTT (Sigma-Aldrich #D0632) was added to each sample and incubation was performed for three instead of two hours to increase the RNA probe yield. The RNA probes were sephadex-purified using the Illustra ProbeQuant G-50 Micro Columns (GE Healthcare Life sciences #28903408; Pittsburgh, USA) and precipitated overnight at − 20 °C (4 M LiCl, Sigma-Aldrich #L7026; 96–100% ethanol). Precipitated probes were washed twice for 15 min each in 70% ethanol, air-dried at room temperature, and dissolved in 20 µl nuclease-free water (Thermo Fisher Scientific #R0581). The probes were stored at − 80 °C.

### In situ hybridisation

Fixed and stored *Acanthochitona fascicularis* specimens were incubated in EGTA in methanol (90% methanol; 0.05 M EGTA pH 8). Subsequently, the EGTA solution was stepwise exchanged by an ascending (20%, 50%, 50%, 80%, 100%) phosphate buffered saline series (Roth #1058.1) with 0.1% Tween20, (Roth #9127.1; PBT). Specimens were then incubated for a maximum of 2 h in PPE (PBT; 0.05 M EGTA pH 8; 4% PFA) for decalcification and were subsequently washed thrice for 10 min each in PBT. Specimens were incubated in a solution of 50 µg/ml proteinase-K in PBT (Roche #03115879001) for 10 min at 37 °C and then washed twice for 5 min each and twice for 10 min each in PBT at room temperature. In order to reduce charged probe binding, specimens were subsequently incubated for 10 min each in 1% triethanolamine (PBT with 1% TEA added; Sigma-Aldrich #90279), for 5 min each in 1% TEA with 0.15% acetic anhydride (Prolabo #21390293; Bern, Switzerland), and for 5 min each in 1% TEA with 0.3% acetic anhydride added. Specimens were then washed twice for 5 min each and twice for 10 min each in PBT and post-fixed in 4% PFA for 45 min. Afterwards, the specimens were washed twice for 5 min each and twice for 10 min each in PBT and were incubated in hybridization buffer (50% formamide, Roth #P040; 5× saline sodium citrate SSC, Roth #10541; 100 µg/ml heparin, Sigma-Aldrich #H3149; 5 mM EDTA, Roth #80401; Denhardt’s block reagent, Sigma-Aldrich #D2531; 100 µg/ml yeast tRNA, Sigma-Aldrich #R675; 0.1% Tween20; 5% dextransulfate, Sigma-Aldrich #D8906) for 10 min at room temperature and additionally for approximately 24 h at 60–62 °C in a water bath. Complementary antisense probes and sense probes (0.5–2 ng/µl) were preheated in 300 µl 100% hybridization buffer for 10 min at 85 °C. One RNA probe per specimen patch was added and hybridization was performed at 60–62 °C for approximately 24 h. Next, the specimens were washed thrice for 20 min each in 4× Wash (50% formamide; 4× SSC; 0.1% Tween20), twice for 20 min each in 2× Wash (50% formamide; 2× SSC; 0.1% Tween20), and twice for 15 min each in 1× Wash (50% formamide; 1× SSC; 0.1% Tween20). Subsequently, specimens were washed thrice for 10 min each in SSCT (1× SSC; 0.1% Tween20) and then washed four times for 10 min each in 0.1 M maleic acid buffer (MAB) (0.1 M MAB pH 7.5 Sigma-Aldrich #M0375; 0.15 M NaCl; 0.1% Tween20). To prevent non-specific anti-digoxigenin antibody binding, specimens were incubated for two hours in 2% MAB block solution (0.08 M MAB, pH 7.5; 2% block reagent #11096176001). Afterwards, specimens were incubated in an anti-digoxigenin antibody conjugated to an alkaline phosphatase enzyme (1:5000; Roche #11093274910) in 2% MAB block solution overnight at 4–7 °C. Alkaline phosphatase enzyme requires a pH of 9.5 to function, thus a respective alkaline phosphatase buffer (AP) was used (0.5 M Tris pH 9.5, Roth #4855.1; 0.5 M NaCl). Next, the specimens were washed four times for 10 min each in PBT and then thrice for 10 min each in alkaline phosphatase buffer (AP; 0.1% Tween20). Signal was developed with a staining buffer (1× AP-buffer; 3.75 µl/ml BCIP, Roche #11383221001; NBT 5 µl/ml, Roche #11383213001) or, alternatively, with a staining buffer that contained 7.5% polyvinyl alcohol (1× AP-buffer without Tween20 but with 75 mg/ml polyvinyl alcohol, Sigma-Aldrich #P1763; 3.75 µl/ml BCIP; NBT 5 µl/ml). Staining time ranged from 20 to 30 min in case of *MHC* and from 3 to 4 h in case of *Mox*, *HESC2*, and *HESC7*. In case of *HESC1* and *HESC3-C6*, staining was additionally performed over a longer time period, ranging from 16 to 23 h, but yielded no signal. Negative controls were performed by following the same in situ hybridization protocol but replacing the antisense probe with its corresponding sense probe. These experiments yielded no signal (for gene phylogenies, see Suppl. Figs. 1–3, for negative controls, see Suppl. Fig. 4).

Signal development was stopped by washing the specimens twice for 5 min each in AP buffer and thrice for 10 min each in PBT. Then, the specimens were post-fixed in 4% PFA for 30 min each and subsequently washed twice for 5 min each and twice for 10 min each in PBT. Specimens were stored in 50% glycerol (Roth #3783.1) diluted in PBT. Prior to clearing, specimens were washed twice for 10 min each in an ascending DEPC series in PBT (20%, 40%, 60%, 80%, 100%) and afterwards twice for 10 min each in an ascending ethanol series in DEPC (20%, 40%, 60%, 80%, 100%). Specimens were mounted on glass slides and cleared in 2:1 benzyl benzoate:benzyl alcohol (Sigma-Aldrich #B9550 and #402834). Specimens were studied with an Olympus BX53 light microscope (Olympus, Tokyo, Japan) and images were taken with a DP73 camera (Olympus). Images were edited with Fiji^44^. Expression pattern schemes were designed with Inkscape (version 0.92.4; https://inkscape.org) and Gimp 2 (Version 2.8.22; https://www.gimp.org).

Between 15 and 40 specimens per gene and developmental stage were investigated in detail for precise location of their expression domains. In almost all cases, 100% of the specimens showed identical expressions patterns. Exceptions to this are *HES2* expression in the gastrula (consistent expression in 20 out of 25 specimens) and in the early larva (25 consistent patterns out of 35 specimens) as well as *HES7* in the early larva (20 consistent expression domains out of 25 specimens). For *HES7* expression experiments in the mid-trochophore stage only five specimens were available, all of which showed identical expression patterns.

### Screening for genes of interest

The publicly available *Acanthochitona fascicularis* translated transcriptome^45^ (erroneously assigned to as *Acanthocithona crinita* therein) was downloaded (https://zoology.univie.ac.at/open-data/) and de-duplicated using cd-hit (Version 4.7), setting the sequence identity threshold to 0.95^46,47^. *Mox* and *MHC* sequences from other mollusks and lophotrochozoans were obtained from the NCBI GenBank database (https://www.ncbi.nlm.nih.gov/) (Supplementary Tables 2, 3, 4) and were used for reciprocal similarity-based searches of the *A. fascicularis* database using the blastp tool (Version 2.8.1+)^48^ with the e-value set to 1e − 6. Protein domain architecture of the resulting *A. fascicularis* candidate sequences was determined using the hmmscan algorithm against the Pfam A database (https://pfam.xfam.org/). In the case of the *HES* genes, a hmm search (Version 3.1b2)^49^ was performed with the *HES* family-specific hairy orange domain as a query (Pfam code: PF07527.13). The hairy orange hmm file (Pfam code: PF07527.13) was downloaded from the Pfam database. Seven *HES* gene candidates turned out to possess a complete basic helix–loop–helix domain, a hairy orange domain, and the WRPW motif, and these were used for further analysis.

### Gene annotation trees

To obtain additional *HES* sequences, the *Crassostrea gigas* Ensembl peptide file^50^ (https://metazoa.ensembl.org/index.html) was queried with hmmsearch (Version 3.1b2) from the HMMER package^49^ using the Pfam HES hidden markov model (Pfamcode: PF07527.13). Using this approach, we identified six *HES* gene candidates that met the threshold e-value of 1e – 3 and also possessed the two complete protein domains typical of *HES* genes (bHLH, Hairy orange, together with the WRPW motif). (Supplementary Table 4). The *Nematostella vectensis* peptide file^51,52^ yielded seven *myosin* sequences (Supplementary Table 2) of a non-bilaterian representative. The myosin head domain hmm file (Pfam code: PF00063.21) was downloaded and used as query for the hmm search. High accuracy multiple sequence alignments were calculated using mafft (Version 7.397)^53^ with the parameters -maxiterate set to 1000 and -localpair. Alignments were trimmed using BMGE (Version 1.12)^54^ by setting the entropy-like value of the blosum matrix to -BLOSUM30, the maximum entropy threshold to 1, and the minimum length of selected regions to 1. The models for amino acid replacement were calculated using prottest (Version 2.1)^55,56^. All available matrices were included (-all-matrices) and models with rate variation among sites (-all-distributions) were included. The likelihood of the predicted models was assessed with the Akaike information criterion (-sort A)^57^. Selected amino acid substitution models were LG^58^ for *MHC* and *HES*, and WAG^59^ for *Mox*. Maximum likelihood trees and Bootstrap analyses (100 bootstraps, -b 100) were performed using phyml (Version 20120412)^60^. Tree topology (t), branch length (l), and rate parameters (r) were optimized (-o tlr). Visualisation and annotation of alignments was performed using aliview (Version 1.0.0.0; https://ormbunkar.se/aliview/)^61^, Jalview (version 2.11.0.; https://www.jalview.org/)^62^, Gimp 2 (Version 2.8.22; https://www.gimp.org), and Inkscape (version 0.92.4; https://inkscape.org). Visualisation and annotation of phylogenetic trees was performed with FigTree (Version 1.4.4.; http://tree.bio.ed.ac.uk/software/figtree/)^63^.

## Results

### Identity of genes of interest

#### Myosin heavy chain (MHC)

One *AfaMHC* ortholog was found in the *Acanthochitona fascicularis* transcriptome^45^ (see Suppl. Fig. 1A). The annotated *AfaMHC* sequence contains one myosin head domain and one myosin tail domain. A *MHC*-specific glycine (peptide sequence: idfGxdl) insertion within the myosin head domain^22^ provides further confirmation of gene identity (Suppl. Fig. 1B). Phylogenetic analysis was performed with eight other members of the myosin superfamily that are commonly found in metazoans (Suppl. Fig. 1A). Myosin members which are specific to given taxa were not included in the analysis^24^. A bootstrap analysis with 100 bootstrap replicates was performed to provide statistical support. *Myosin I* is argued to be an ancient member of the myosin superfamily^24^ and thus was used to root the tree. The annotated *AfaMHC* sequence clusters together with its respective metazoan orthologs. The *MHC* clade is well supported as are the clades of the other myosin family members.

#### Mox

In the *Acanthochitona fascicularis* transcriptome two *Mox* sequences, referred to as *AfaMox1* and *AfaMox2*, were found. *Mox* genes possess a homeodomain with a glutamic acid site that is specific for *Mox* genes (Suppl. Fig. 2B). It shares a common origin with *Evx*, another homeotic gene. *Mox* and *Evx* together form the sister group to the Hox class genes^64^. Bootstrap analysis with 100 replicates supports the clustering of *AfaMox1*, *AfaMox2*, and *AfaEvx* with their orthologs (Suppl. Fig. 2A).

#### Hairy and enhancer of split (HES)

Seven putative *HES* sequences, *AfaHESC1* (“C” is for candidate), *AfaHESC2*, *AfaHESC3*, *AfaHESC4*, *AfaHESC5*, *AfaHESC6*, and *AfaHESC7*, were found in the *Acanthochitona fascicularis* transcriptome. HES proteins belong to the bHLH transcription factors and possess two domains, namely a bHLH domain that contains a *HES* gene-specific proline residue and a Hairy orange domain. In addition, they possess a *HES*-specific WRPW motif at their C-terminal end (Suppl. Fig. 3B). The phylogenetic analysis supports the monophyly of the identified *HES* sequences, which form a sister group relationship to *Hey*-class genes (*Hairy and enhancer of split related with a YRPW motif*, see Suppl. Fig. 3A). These possess the same two domains mentioned above, in addition to the tetrapeptide with a tyrosine instead of a tryptophan at the first position. The third group of genes related to the *HES* family are the *Helt* genes (*Hairy and enhancer of split-related protein Helt*), which only possess the bHLH domain and the Hairy orange domain but lack the specific tetrapeptide at the C-terminal end. The most distantly related gene group, *Clockwork orange*, was used as an outgroup. Similar to *Helt*, Clockwork orange only possesses the bHLH and the Hairy orange domain.

### *MHC* is expressed in all larval and most adult muscle systems

**Figure 1:**
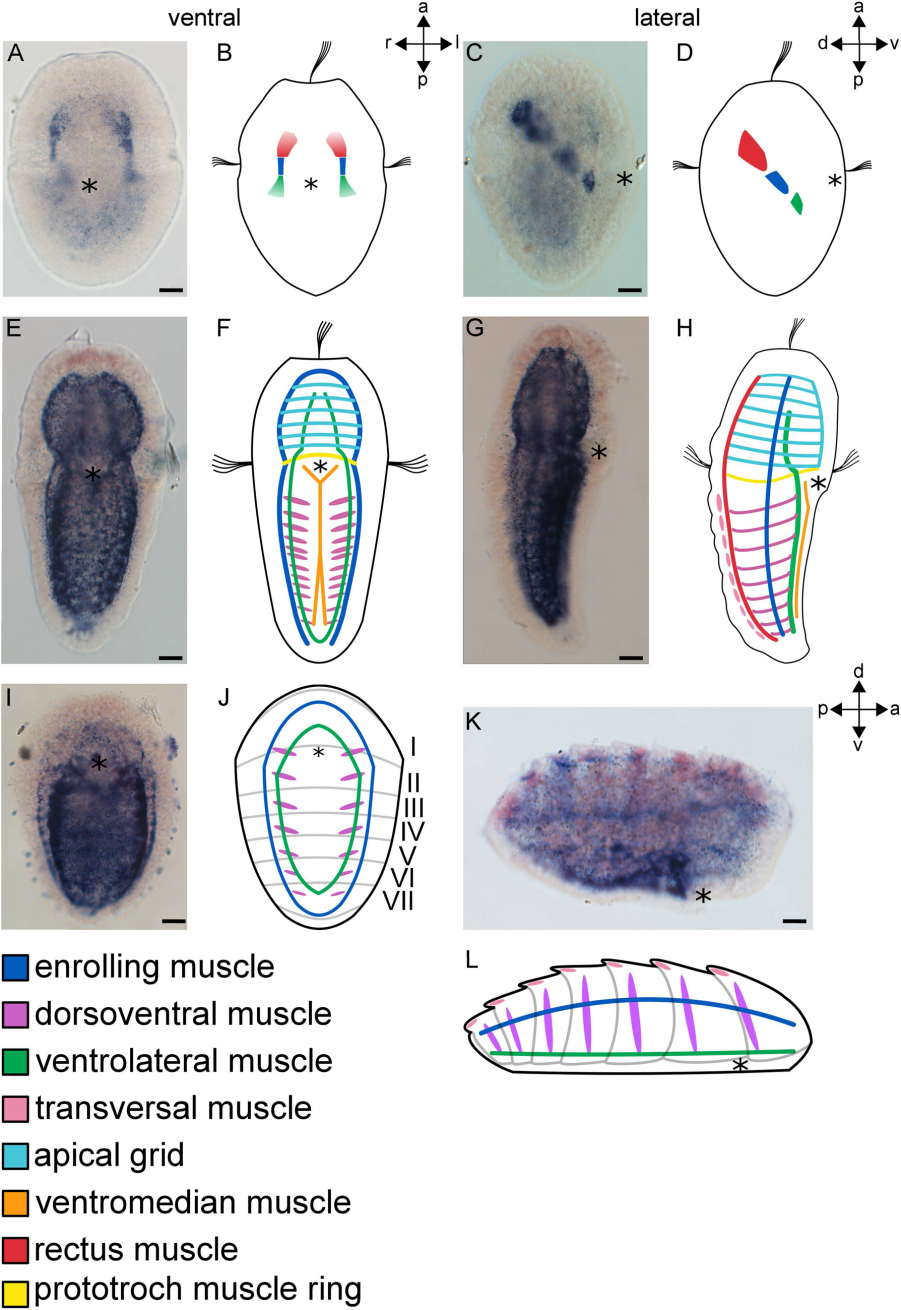
Expression of *AfaMHC* during *Acanthochitona fascicularis* development. (**B**, **D**, **F**, **H**, **J**, **L**) are schematic representations of gene expression signatures of the respective developmental stages. Colour code indicates respective muscle systems. (**A**–**D**) *AfaMHC* expression in the early trochophore larva (**A**) *AfaMHC* expression in the developing rectus, enrolling, and ventrolateral muscles. (**B**) Ventral view of the developing muscles. (**C**) Lateral right view of the *AfaMHC* expression in developing muscles. (**D**) Lateral right view. (**E**–**H**) *AfaMHC* expression in the late trochophore larva. (**E**) *AfaMHC* expression is found in all muscles. Dorsally located muscles such as the rectus muscle and the transversal muscles are partially masked by the intense staining of the more ventrally positioned muscles. (**F**) Ventral view. Rectus and transversal muscles are not shown. (**G**) Lateral view showing weak expression in the transversal muscles. (**H**) Lateral right view. (**I**–**L**) *AfaMHC* expression in the early juvenile. (**I**) *AfaMHC* expression is retained in the enrolling muscle, the ventrolateral muscle, the dorsoventral muscles, and the transversal muscles. (**J**) Ventral view. (**K**) Lateral right view of *AfaMHC* expression. (**L**) Lateral right view. Asterisks mark the mouth. Roman numbers correspond to the future juvenile shells. *a* anterior, *d* dorsal, *l* left, *p* posterior, *r* right, *v* ventral. Scale bars equal 20 µm. Expression pattern schemes were designed with Inkscape (version 0.92.4; https://inkscape.org) and Gimp 2 (Version 2.8.22; https://www.gimp.org).

Expression of *AfaMHC* was first detected in early trochophore stages during muscle formation (Fig. 1A–D). *AfaMHC* is expressed in three small, paired regions which give rise to the rectus muscle that spans the region below the future shell plates in anterior–posterior direction. In addition, expression is in the enrolling muscle that laterally engulfs the larva and the ventrolateral muscle that lies ventrally and consists of two longitudinal muscle strands (see^39,65^ for detailed description of polyplacophoran larval myoanatomy) (Fig. 1A–D). In the late trochophore larva, all larval muscle systems (i.e., muscles that do not persist until adulthood) are labelled (Fig. 1E–H), including the prototroch muscle ring that underlies the prototroch, the paired ventrolateral muscle, the single ventromedian muscle, and the apical muscle grid^39,65^. Muscles that are maintained and elaborated after metamorphosis are the enrolling muscle, the dorsal longitudinal rectus muscle, seven sets of paired dorsoventral muscles (with the eighth being formed only considerably later during post-metamorphic development), and a set of dorsal transversal muscles that underlie the shell plates^39,65^. Of these, expression of *AfaMHC* is in the enrolling muscle, the rectus muscle, and the dorsoventral muscles (Fig. 1E–H). Relatively weak expression domains are found in the region of the developing dorsal transversal muscles (Fig. 1G,H). In the juvenile polyplacophoran, the larval muscles disappear and the muscles of the future adult body plan become elaborated. Accordingly, individual myocytes become concentrated into distinct sets of dorsoventral and transversal muscles. Adult-specific muscles, such as the buccal musculature that forms several strands around the mouth, and the paired radula retractors develop^39,65^. The ventrolateral muscle is still partly visible at this point and is reduced during further growth. Of these juvenile muscle systems, *AfaMHC* expression is found in the ventrolateral muscle, the enrolling muscle, the dorsoventral muscles, and in the transversal muscles (Fig. 1I–L).

### *Mox* is expressed in the mesodermal bands and in a subset of the musculature

**Figure 2:**
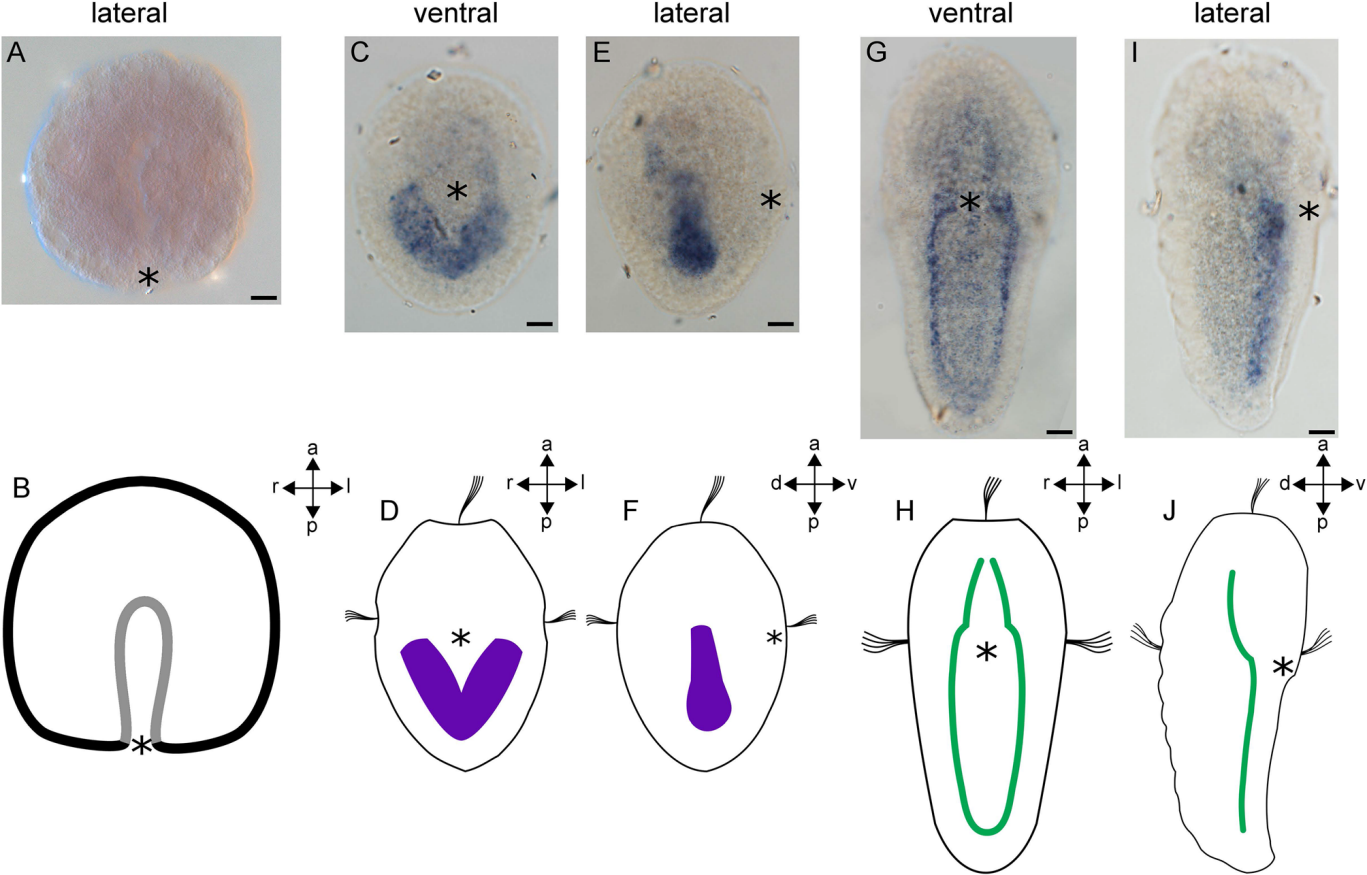
Expression of *AfaMox1* during early mesoderm formation in *Acanthochitona fascicularis*. (**B**, **D**, **F**, **H**, **J**) are schematic representations of gene expression patterns of the respective developmental stages with gene expression domains indicated in purple and the ventrolateral muscle in green. (**A**) The gastrula is devoid of *AfaMox1* expression. (**B**) Lateral right view. (**C**–**F**) *AfaMox1* expression in the early trochophore larva. (**C**) *AfaMox1* is expressed in the mesodermal bands. (**D**) Ventral view. (**E**) Lateral right view of *AfaMox1* expression in the mesodermal bands. (**F**) Lateral right view. (**G**–**J**) *AfaMox1* expression in the late trochophore larva. (**G**) *AfaMox1* expression in the ventrolateral muscle. (**H**) Ventral view. (**I**) Lateral right view of *AfaMox1* expression in the ventrolateral muscle. (**J**) Lateral right view. Asterisks mark the blastopore and the mouth, respectively. *a* anterior, *d* dorsal, *l* left, *p* posterior, *r* right, *v* ventral. Scale bar equals 20 µm. Expression pattern schemes were designed with Inkscape (version 0.92.4; https://inkscape.org) and Gimp 2 (Version 2.8.22; https://www.gimp.org).

Of the two *Mox* sequences identified we were only able to produce expression data by in situ hybridisation for *AfaMox1*. Expression of this gene was first detected in the early trochophore larva (Fig. 2A,B), where it is prominently expressed in the developing paired mesodermal band (Fig. 2C–F). In the late trochophore larva, *AfaMox1* expression is confined to the ventrolateral muscle (Fig. 2G–J). No *Mox* expression was detected in later stages of development.

### *HES* genes are expressed in ectodermal and mesodermal domains

**Figure 3:**
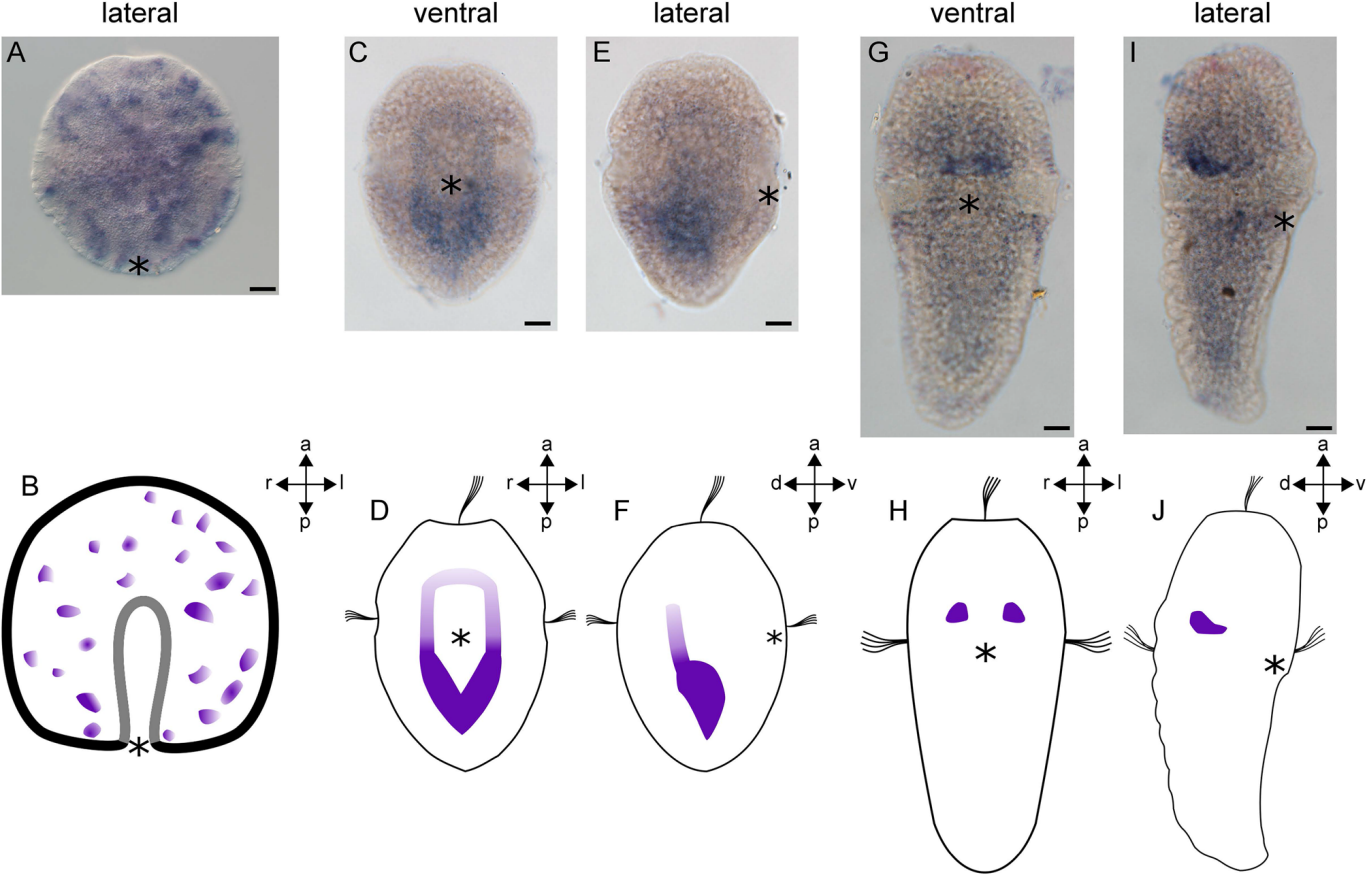
Expression of *AfaHESC2* during early mesoderm formation in *Acanthochitona fascicularis*. (**B**, **D**, **F**, **H**, **J**) are schematic representations of gene expression signatures (in purple) of the respective developmental stages. (**A**, **B**) Expression of *AfaHESC2* in the gastrula. (**A**) *AfaHESC2* is expressed in ectodermal cells of the gastrula. (**B**) Lateral view (**C**–**F**) *AfaHESC2* expression in the early trochophore larva. (**C**) *AfaHESC2* is expressed in the mesodermal bands. A weak expression domain extends into the larval episphere. (**D**) Ventral view. (**E**) Lateral right view of *AfaHESC2* expression in the early trochophore larva. A weak expression domain extends into the larval episphere. (**F**) Lateral right view. (**G**–**J**) *AfaHESC2* expression in the late trochophore larva. (**G**) *AfaHESC2* is expressed in two spot-like domains in the region of the adult buccal ganglia. (**H**) Ventral view. (**I**) Lateral view of *AfaHESC2* expression showing that the spot-like expression domains are located dorsally. (**J**) Lateral view. Asterisks mark the blastopore and the mouth, respectively. *a* anterior, *d* dorsal, *l* left, *p* posterior, *r* right, *v* ventral. Scale bar equals 20 µm. Expression pattern schemes were designed with Inkscape (version 0.92.4; https://inkscape.org) and Gimp 2 (Version 2.8.22; https://www.gimp.org).

**Figure 4:**
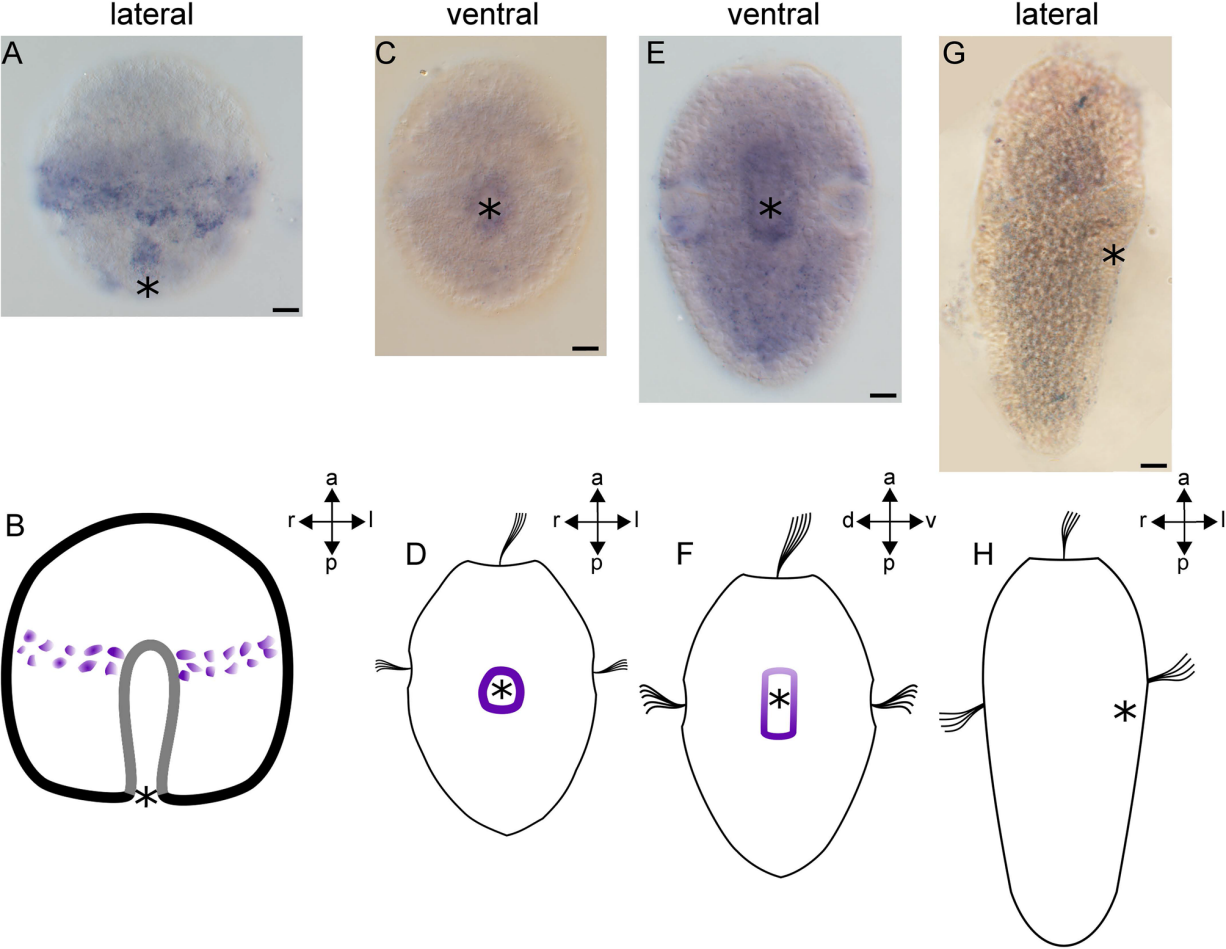
Expression of *AfaHESC7* during development of *Acanthochitona fascicularis*. (**B**, **D**, **F**, **H**) are schematic representations of gene expression signatures (in purple) of the respective developmental stages. (**A**, **B**) Expression of *AfaHESC7* in the gastrula. (**A**) *AfaHESC7* is expressed in the trochoblasts. (**B**) Lateral view. (**C**, **D**) *AfaHESC7* expression in the early trochophore larva. (**C**) Expression of *AfaHES7* is found in ectodermal cells around the mouth. (**D**) Ventral view. (**E**, **F**) Expression of *AfaHESC7* in the mid-trochophore larva. (**E**) The expression domain remains around the posterior margin of the mouth and extends anteriorly into the region of the foregut. (**F**) Ventral view. (**G**) Loss of *AfaHESC7* expression in the late trochophore larva. (**H**) Lateral right view. Asterisks mark the blastopore and the mouth, respectively. *a* anterior, *d* dorsal, *l* left, *p* posterior, *r* right, *v* ventral. Scale bar equals 20 µm. Expression pattern schemes were designed with Inkscape (version 0.92.4; https://inkscape.org) and Gimp 2 (Version 2.8.22; https://www.gimp.org).

Two of the seven HES family genes identified (*AfaHESC2* and *AfaHESC7*) yielded expression signals. Both genes start to be expressed in the late gastrula stage. Their expression is maintained in early larval stages but only *AfaHESC2* is expressed in the late trochophore larva. In the gastrula, *AfaHESC2* is expressed in ectodermal cells (Fig. 3A,B). In the early trochophore larva, *AfaHESC2* is expressed in the mesodermal bands. A weaker expression domain extends from the anterior pole of the mesodermal bands into the apical region of the larva where it closes in an inverted U-shaped manner (Fig. 3C–F). In the late trochophore larva, *AfaHESC2* expression is limited to the region of the adult buccal ganglion close to the dorsal ectoderm, where two spot-like expression domains are located (Fig. 3G–J). Expression of *AfaHESC7* first occurs in the prospective trochoblasts in the equatorial region of the gastrula (Fig. 4A,B). In the early larval stage, *AfaHESC7* expression is restricted to a domain around the mouth (Fig. 4C,D). Throughout larval development, *AfaHESC7* expression continues to be expressed around the mouth and in the region of the presumptive foregut. *AfaHESC7* expression ceases in the late trochophore larva (Fig. 4E–H).

## Discussion

### *Myosin heavy chain*: a conserved marker of metazoan myogenesis

Results from the cnidarian *Nematostella*^25^ suggest that *myosin heavy chain* (*MHC*) was already a key component of contractile cells in the last common ancestor of cnidarians and bilaterians. *MHC* has been used as a marker to study early muscle differentiation across lophotrochozoans^66–68^, ecdysozoans^18,69,70^, and deuterostomes^28,29,71^. Consistent with these data, *MHC* is expressed during the early formation of several muscle systems in *Acanthochitona* larvae, including the ventrolateral muscle, the enrolling muscle, and the rectus muscle. These results confirm the utility of *MHC* as a marker of early myogenesis in Mollusca, although further studies are needed to allow for a more detailed comparison of the initial stages and domains of muscle differentiation in this phylum.

### Conserved *Mox* expression in nephrozoan mesoderm and muscle formation

Most metazoans possess only one *Mox* gene^3,7,13,14,33,72–74^ with exception of the vertebrates that have two^31,32^ and the anthozoan *Nematostella vectensis* that has four *Mox* genes that evolved by tandem duplications^75^. Cnidarian *Mox* genes are expressed exclusively in the endoderm^73,74,76^, whereas in most bilaterians, *Mox* expression initially coincides with mesoderm formation and is later restricted to the developing musculature^7,72^.

In deuterostomes, *Mox* expression typically begins around the time of gastrulation in early mesodermal precursors. In the hemichordate *Saccoglossus kowalevskii*, *Mox* (*SkoMox*) is expressed in the ventral mesoderm during formation of the paired coelomic cavities of the metasome^14^. Data are inconclusive as to whether or not *SkoMox* expression continues during subsequent development^14^. In the ascidian *Ciona intestinalis*, the *Mox* ortholog *Meox* (*CinMox*) is specifically expressed in muscle precursor cells in the early gastrula^77^ and in the cephalochordate *Branchiostoma floridae*, *BbeMox* is expressed in the paraxial mesoderm during somite formation^13^. In the mouse, two *Mox* genes, *MmuMox1* and *MmuMox2*, were identified. These show slightly different expression dynamics and have been implicated in the early anterior–posterior patterning of the embryonic mesoderm as well as in somite specification and differentiation^32^. A reduction of limb muscle tissue in *MmuMox2* null mice revealed the importance for muscle development^78^. A *Mox* mutation in zebrafish causes defects in bone development such as vertebral fusion, congenital scoliosis, and asymmetry of the pectoral girdle, providing evidence for the involvement of *Mox* in establishing mesodermal derivatives^79^. These data imply a conserved involvement of *Mox* in the initial specification of the deuterostome mesoderm and in the development of its derivatives.

In the diverse Lophotrochozoa, *Mox* expression has only been studied in three species, namely the gastropod *Haliotis asinina*^72^, the brachiopod *Terebratalia transversa*^3^, and the polychaete *Alitta virens*^7^. For each of these, only one *Mox* gene has been described, while we found a second *Mox* sequence in the polyplacophoran *Acanthochitona fascicularis*. All four species start to express *Mox* shortly after gastrulation in lateral mesodermal bands that flank the endoderm. Accordingly, an early role for *Mox* in mesodermal band specification appears to be an ancestral feature of lophotrochozoans. During later stages, *Mox* continues to be expressed in the developing foot musculature in *Haliotis*^72^, in precursor cells of the future body wall and pharyngeal muscles of *Alitta*^7^, and in the ventrolateral muscle of late *Acanthochitona* trochophore larvae. Since we were not able to produce consistent expression results for *AfaMox2*, a putative role of this gene remains speculative. However, taken together, these data support a dual role of *Mox* in early mesoderm specification and in myogenesis, that is conserved among lophotrochozoans and deuterostomes. Notably, however, several lineage-specific evolutionary events have resulted in the loss of conserved roles and in co-option of *Mox* into novel ones. The *Mox* ortholog of the sea urchin *Strongylocentrotus purpuratus*, for example, is not expressed during mesoderm formation but in ectodermal neurons in the region of the larval apical organ^80^. This expression disappears in later stages, indicating that *SpuMox* plays a role in early neurogenesis rather than in mesoderm or muscle formation^80^. A similar situation is found in the fruit fly *Drosophila melanogaster*, where the *Mox* ortholog *buttonless* (*DmeMox*) is expressed in the dorsal median cells which derive from the ventral mesoderm and play a crucial role in axon guidance. Importantly, however, *DmeMox* is not expressed in muscle progenitors or muscular tissue^33^. In the second major ecdysozoan lineage, Nematoda, *Mox* was very likely lost altogether^81^. Since other ecdysozoans and echinoderms are yet to be tested for *Mox* expression, a potential association between the loss of mesodermal *Mox* expression and the evolution of a neurogenesis-related role remains uncertain.

**Figure 5:**
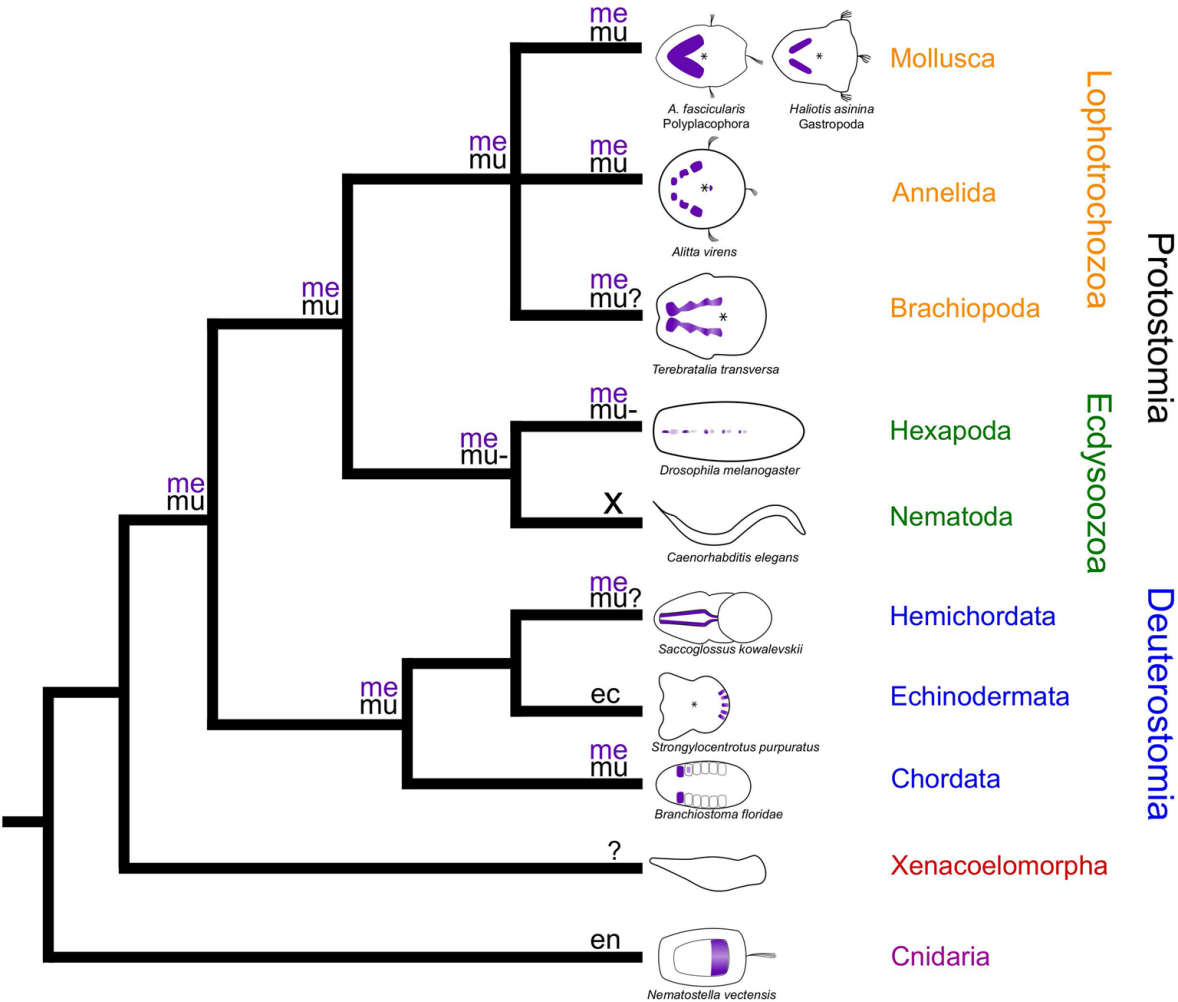
Comparative *Mox* expression in eumetazoans. Mesodermal domains of *Mox* expression in purple in schematic representations. Schemes are in ventral view with anterior to the right. ec = expression in ectoderm, en = expression in endoderm, me = expression in mesoderm, mu = *Mox* expression in developing muscles, mu- = no *Mox* expression in developing muscles, mu? = *Mox* expression in developing muscles not investigated, x = no *Mox* ortholog present. Lophotrochozoa: *Mox* is expressed in the mesodermal bands of early lophotrochozoan larvae and additionally in a small pre-oral ectomesodermal domain in *Alitta virens*. *Mox* is also expressed in muscle progenitor cells and/or muscle tissue in later-stage mollusk and annelid larvae. Data on brachiopods are inconclusive. Ecdysozoa: the *Mox* ortholog *buttonless* is expressed in dorsal median cells in *Drosophila* which originate from the mesoderm and play a role in axon guidance but are not associated with myogenesis. Nematodes have no *Mox* ortholog. Deuterostomia: *Mox* expression in the mesoderm in hemichordates and chordates. In the sea urchin, *Mox* is only expressed in neural cells of the larva. *Mox* expression in myogenesis in hemichordates is unknown. In chordates, *Mox* is expressed during somitogenesis in amphioxus and vertebrates. In amphioxus, no *Mox* expression was observed after somitogenesis. In vertebrates, both *Mox* genes are expressed in myogenesis. Xenacoelomorpha: No unambiguous *Mox* ortholog described. Cnidaria: *Mox* expression is restricted to the endoderm. Parsimony analysis suggests recruitment of *Mox* in mesoderm formation and myogenesis at the base of bilaterians with a loss in myogenesis in *Drosophila* and a loss in mesoderm formation in echinoderms. Asterisks mark the mouth. Data from previous investigations^3,7,13,14,33,72,76,78,79^ and present study. Expression pattern schemes were designed with Inkscape (version 0.92.4; https://inkscape.org) and Gimp 2 (Version 2.8.22; https://www.gimp.org).

In summary, the data currently available suggest that *Mox* was recruited into mesoderm formation in the last common bilaterian ancestor (LCBA) and may thus have played an important role in mesoderm evolution (Fig. 5). In addition, it appears that *Mox* was simultaneously recruited into myogenesis in the LCBA with loss of this role at least in *Drosophila* and putatively in both, myogenesis and mesoderm formation, in echinoderms (Fig. 5).

### Variability of *HES* gene expression in metazoan development

*HES* genes are fast evolving genes that have undergone repeated species-specific, independent gene duplications^37^. The actual number of *HES* copies varies from one single sequence in the cnidarian *Hydra*^36^, the leech *Helobdella*^82^, the fly *Drosophila*^83^, and the sea urchin *Strongylocentrotus*^84^ to up to 22 copies in the zebrafish *Danio*^37^. In *Acanthochitona fascicularis*, seven *HES* genes were identified, and two (*AfaHESC2* and *AfaHESC7*) were further investigated here by in situ hybridization.

*HES* genes have been implicated in a wide range of developmental processes including neurogenesis as well as digestive tract and mesoderm formation. Thus, *HES* expression domains vary considerably between taxa. A comparative overview of the identified *Mox*, *HES*, and *MHC* genes and their respective expression domains across Metazoa is provided in Supplementary Table 5. In the sea anemone *Nematostella*, two *HES* genes, *NveHES2* and *NveHES3,* are expressed in ectodermal cells of the gastrula, while *NveHES3* expression expands to oral ecto- and endoderm in the planula larva^85^. In contrast, the single *Hydra HES* gene (*HvuHES*) is expressed during budding at the bud base shortly before separation from the mother animal, but was not detected in earlier stages^36^. In early embryos of the acoelomorph *Symsagittifera roscoffensis*, the only *HES* gene, *SroHES*, is expressed in the anterior-median region. In juveniles, it is expressed posterior to the statoblast, dorsally in the nerve cords, and mid-ventrally in the brain, but not in the mesoderm^86^. These data indicate that *HES* genes were initially involved in neurogenesis and in development of anterior ecto- and endodermal tissues and that their mesodermal expression might be a nephrozoan (or even bilaterian) novelty.

Deuterostomes, such as the cephalochordate *Branchiostoma*, and vertebrates possess multiple *HES* genes that are broadly expressed across all germ layers. In *Branchiostoma*, four out of eight *HES* genes (*BbeHESA-D*) are expressed in the anterior endoderm, in the presumptive neural plate, and in the presomitic mesoderm of the mid-gastrula^87^. In neurula stages, expression is further found in the endoderm, in the neural tube, in the somites, as well as in the paraxial mesoderm, the foregut, the neural plate, and in the notochord^87^. In vertebrates (mouse, chicken, and *Xenopus*), *HES* genes also play a crucial role during somitogenesis, gut formation, neurogenesis, as well as in the maintenance of stem cell potential and separation of different brain areas from each other^35,88,89^. A functional study employing *HES* gene knockdown in *Xenopus laevis* resulted in a decrease of cell proliferation. This indicates anti-apoptotic functions and highlights the ability for transcriptional repression of *HES* genes^89^. In the sea urchin *Strongylocentrotus* on the other hand, no mesodermal expression of *HES* was observed^84^. Instead, *HES* is expressed from blastula to gastrula stages in the oral ectoderm and (weakly) in the archenteron^84^. This is consistent with data on *SpuMox* that, in contrast to *Mox* genes of other deuterostomes, is also absent from the mesoderm and is exclusively expressed in ectodermal neurons in the sea urchin^80^.

Interestingly, *Mox* and *HES* genes also seem to be of relatively little importance for mesoderm development and myogenesis in ecdysozoans^33,81,90^. In the nematode *Caenorhabditis elegans*, *ref-1* (*CelHES*) is only expressed in descendants of the AB blastomere, which contribute to the nervous system^91^. The single *Drosophila HES* gene, *hairy* (*DmeHES*), is mainly expressed ectodermally during segmentation, where it acts as a pair-rule gene^90^. In later stages, *DmeHES* is also expressed in the nervous system, the foregut, the tracheal primordia and surrounding mesoderm, as well as in somatic and visceral muscles^92,93^. However, *DmeHES* does not seem to affect myogenesis, but rather contributes to tracheal development^93^.

Lophotrochozoan *HES* gene expression is highly species-specific and has been described across all germ layers. The single planarian *HES* gene is exclusively expressed in neuronal progenitor cells^94^, whereas the three and 13 *HES* genes of the annelids *Platynereis* and *Capitella*, respectively, are expressed across various body regions, including the growth zone, the chaetae, the nervous system, and the digestive tract^19,37^. In the brachiopod *Terebratalia*, *TtrHES1* is only transiently expressed in the lateral ectoderm of the gastrula^21^, while *TtrHES2* is expressed in the mesoderm and in the developing chaete but not during formation of the gut^21^. This is similar to the gastropod *Crepidula*, where two *HES* genes, *HESA (CfoHES1*) and *HESB* (*CfoHES2*), are predominantly expressed in ectodermal cells around the blastopore (*CfoHES2*) and mouth (*CfoHES1*). In addition, *CfoHES2* is expressed in ventral neurosensory cells and, during further development, in the anlage of the foot^38^. In contrast to both, the brachiopod and the gastropod, *HES* gene expression was absent during myogenesis in the polyplacophoran *Acanthocitona*. A potential reason for this is that only two out of seven *HES* genes were detectable by in situ hybridization during *Acanthochitona* ontogeny. While in-situ hybridization sensitivity is high, it is possible that one or more of the remaining five *HES* genes are indeed involved in myogenesis but did not meet the minimum expression threshold required for detection. Although we were unable to unequivocally assign *AfaHESC2* expression to distinct morphological features, it is briefly expressed in the mesodermal bands and later appears to overlap with the region of the developing buccal ganglia^95^. *AfaHESC7* expression was observed in the oral ectoderm, around the foregut, and, surprisingly, in the trochoblasts. The latter are specialized founder cells that give rise to the ciliated cells of the prototroch and have so far not been reported to express a *HES* gene in any other lophotrochozoan.

**Figure 6:**
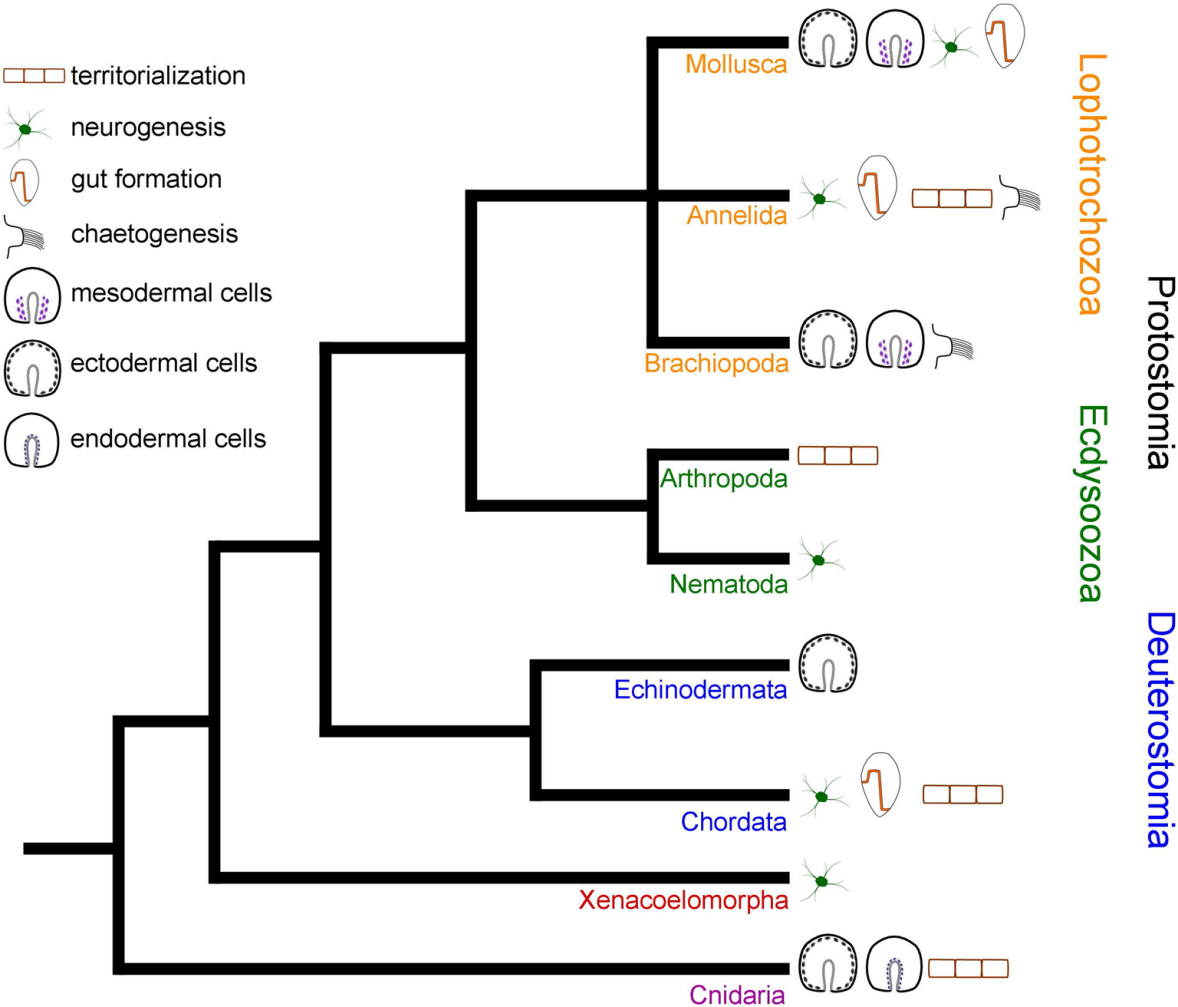
*HES* gene expression in metazoan organogenesis. Lophotrochozoa: Mollusca: Expression is in ectodermal cells of pre-larval stages and subsequently during mesoderm formation as well as in neurogenesis and development of the digestive tract. Annelida: Expression is during formation of the digestive tract, neurogenesis, segmentation, and chaetogenesis. Brachiopoda: Expression is in the ectoderm of pre-larval stages, during early mesoderm formation, and in chaetogenesis. Ecdysozoa: Hexapoda: Expression is during segment formation. Nematoda: Expression is during neurogenesis. Deuterostomia: Echinodermata: Expression is in the larval ectoderm. In the late pluteus larva, *HES* expression is in the region of the apical organ. Chordata: Expression is during neurogenesis, somitogenesis, and in the digestive tract. Xenacoelomorpha: Expression is during neurogenesis. Cnidaria: Expression is in the ectoderm and endoderm of early developmental stages and during budding in hydrozoans. Data from previous investigations^19,21,35–38,81–85^ and present study. Expression pattern schemes were designed with Inkscape (version 0.92.4; https://inkscape.org) and Gimp 2 (Version 2.8.22; https://www.gimp.org).

Taken together, these data show that mesodermal and muscular *HES* gene expression is likely an ancestral feature of bilaterians that was lost in multiple lineages including echinoderms, nematodes, planarians, and possibly also acoelomorphs and polyplacophoran mollusks. Involvement in endoderm specification, on the other hand, likely emerged in the last common ancestor of Metazoa and was also lost in several lineages, such as acoelomorphs, nematodes, planarians, and brachiopods. Altogether, ectodermal and/or neural *HES* gene expression appears to be particularly well conserved across metazoans. Since *HES* genes chiefly act in separating tissues from each other that are destined to undergo fate determination (“territorialisation”), they have been co-opted into various additional developmental processes, such as the formation of the chaete in annelids and brachiopods, segmentation in annelids and arthropods, somitogenesis in chordates, and budding in cnidarians. This enormous variability highlights their importance for the evolution of distinct ontogenetic pathways throughout the animal kingdom (Fig. 6, Supplementary Table 5).

## Conclusion

The present study shows that *Mox* and *HES* genes are expressed during mesoderm formation in the mollusk *Acanthochitona fascicularis*. Expression of *Mox* in the mesodermal bands and in their major derivatives, the muscles, is congruent with the situation in other lophotrochozoans, suggesting a dual role of this gene in the last common bilaterian ancestor. *Mox* experienced loss in myogenesis in ecdysozoans and loss in both myogenesis and mesoderm formation in echinoderms, where it is instead expressed in the ectoderm. Expression of *HES* occurs during early mesoderm development, neurogenesis, and digestive tract formation in a number of bilaterians as well as in ectodermal and endodermal domains in cnidarians, implying either a wide variety of roles already at the dawn of bilaterian evolution or a particularly high degree of variability (co-option) of *HES* genes with various independent gain-of-function events along individual bilaterian lineages.

## Supplementary Information

Supplementary Information.
