# Supplementary material for: HES and Mox genes are expressed during early mesoderm formation in a mollusk with putative ancestral features
Source: Sci Rep. 2021 Sep 9;11:18030. doi: 10.1038/s41598-021-96711-y (PMC8429573; doi:10.1038/s41598-021-96711-y)
Supplement: Supplementary file 1 — Supplementary Information. [file 41598_2021_96711_MOESM1_ESM.pdf]

HES and *Mox* genes are expressed during early mesoderm formation in a mollusk with putative ancestral features

Attila Sachslehner[1], Elisabeth Zieger[1], Andrew Calcino[1], Andreas Wanninger[1,*]

[1] Department of Evolutionary Biology, Unit for Integrative Zoology, University of Vienna, Althanstrasse 14, 1090 Vienna, Austria

* Author for correspondence. Email: andreas.wanninger@univie.ac.at

ORCID IDs:

Attila Sachslehner: 0000-0003-1447-9254

Elisabeth Zieger: 0000-0003-3266-834X

Andrew D. Calcino: 0000-0002-3956-1273

Andreas Wanninger: 0000-0002-3266-5838

Supplementary Information

Supplementary Figures

Supplementary Figure 1. ***Myosin heavy chain*** **phylogeny and alignment.** Nine members of the myosin superfamily that are commonly found in metazoans were included in the analysis. **(A)** Maximum likelihood tree with 100 bootstrap replicates. *AfaMHC* groups with high statistical support with orthologs of other lophotrochozoans. The *Myosin I* clade was used to root the tree. **(B)** Trimmed alignment of the myosin head domain. The *MHC*-specific glycine insertion is indicated by the black arrowhead. Missing data are indicated by dashes. Scalebar indicates substitutions per site. Visualisation and annotation of alignments was performed using aliview (Version 1.0.0.0; https://ormbunkar.se/aliview/)[1], Jalview (version 2.11.0.; https://www.jalview.org/)[2], Gimp 2 (Version 2.8.22; https://www.gimp.org) and Inkscape (version 0.92.4; https://inkscape.org). Visualisation and annotation of phylogenetic trees was performed with FigTree (Version 1.4.4.; http://tree.bio.ed.ac.uk/software/figtree/)[3].

Supplementary Figure 2. ***Mox*** **phylogeny and alignment.** *Evx* was included because it is argued to form the sister group to *Mox* and the *Hox* genes were included because they form the sister group to the *Evx*-*Mox* clade. **(A)** Maximum likelihood tree with 100 bootstrap replicates. *AfaMox1* and *AfaMox2* group with high statistical support with orthologs of other lophotrochozoans. The clade of anterior *Hox* genes was used to root the tree. **(B)** Trimmed alignment of the *Mox* homeodomain. The *Mox*-specific glutamic acid residue is indicated by the black arrowhead. Missing data are indicated by dashes. Scale bar indicates substitutions per site. Visualisation and annotation of alignments was performed using aliview (Version 1.0.0.0; https://ormbunkar.se/aliview/)[1], Jalview (version 2.11.0.; https://www.jalview.org/)[2], Gimp 2 (Version 2.8.22; https://www.gimp.org) and Inkscape (version 0.92.4; https://inkscape.org).

Visualisation and annotation of phylogenetic trees was performed with FigTree (Version 1.4.4.; http://tree.bio.ed.ac.uk/software/figtree/)[3].

Supplementary Figure 3. *Hairy and enhancer of split* phylogeny and alignment. *Hey*, *Helt*, and *Clockwork orange* were included in the analysis because they are the closest relatives of the *HES* genes. **(A)** Maximum likelihood tree with 100 bootstrap replicates. *AfaHES* copies group with high statistical support with HES gene copies of other lophotrochozoans. The *Clockwork orange* clade was used to root the tree. **(B)** Trimmed alignment of the *HES* gene sequences. *HES* genes consist of two domains, namely a bHLH domain that contains a *HES*-specific proline residue (arrowhead) and a *HES*-specific Hairy orange domain, as well as one *HES*-specific WRPW motif at the C-terminal end. Missing data are indicated by dashes. Scale bar indicates substitutions per site. Visualisation and annotation of alignments was performed using aliview (Version 1.0.0.0; https://ormbunkar.se/aliview/)[1], Jalview (version 2.11.0.; https://www.jalview.org/)[2], Gimp 2 (Version 2.8.22; https://www.gimp.org) and Inkscape (version 0.92.4; https://inkscape.org). Visualisation and annotation of phylogenetic trees was performed with FigTree (Version 1.4.4.; http://tree.bio.ed.ac.uk/software/figtree/)[3].

Supplementary Figure 4. Examples of positive in situ hybridization signal using antisense probes compared to negative controls (sense probes) devoid of signal. Same-stage individuals were used for each gene. **(A)** *AfaHESC2* is expressed in ectodermal cells of the gastrula. **(B)** Lack of signal in the control using the respective sense probe. **(C)** *AfaMox1* is expressed in the mesodermal bands in the early trochophore larva. **(D)** Lack of signal in the control using the respective sense probe. **(E)** Expression of *AfaHESC7* around the posterior margin of the mouth and in the region of the foregut. **(F)** Lack of signal in the control using the respective sense probe. Asterisks mark the blastopore and the mouth, respectively.

Abbreviations: a = anterior, l = left, p = posterior, r = right. Scale bar equals 20 µm. Images were designed with Inkscape (version 0.92.4; https://inkscape.org) and Gimp 2 (Version 2.8.22; https://www.gimp.org).

Supplementary Tables

Supplementary Table 1. Primers used for gene amplification. F = forward, R = reverse.

Supplementary Table 2. Species list and GenBank accession numbers used for the *Myosin heavy chain* phylogeny.

Supplementary Table 3. Species list and GenBank accession numbers used for the *Mox* phylogeny.

Supplementary Table 4. Species list and GenBank accession numbers used for the *hairy and enhancer of split* phylogeny.

Supplementary Table 5. Number of investigated genes and their expression domains of the Mox, HES, and MHC families in metazoan phyla.

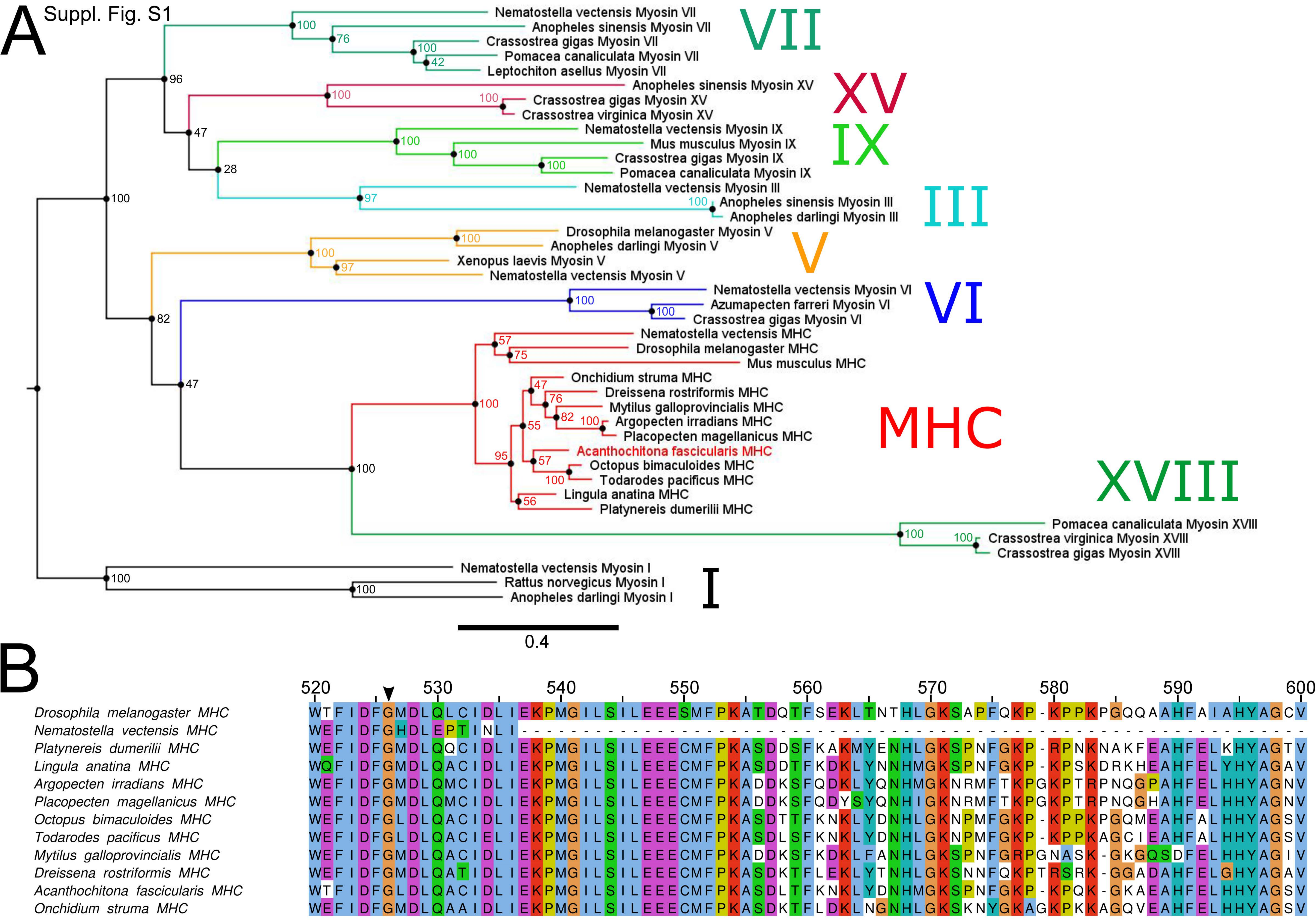
A
Suppl. Fig. S1
Nematostella vectensis Myosin VII
Anopheles sinensis Myosin VII
Crassostrea gigas Myosin VII
Pomacea canaliculata Myosin VII
Leptochiton asellus Myosin VII
VII
Anopheles sinensis Myosin XV
Crassostrea gigas Myosin XV
Crassostrea virginica Myosin XV
XV
Nematostella vectensis Myosin IX
Mus musculus Myosin IX
Crassostrea gigas Myosin IX
Pomacea canaliculata Myosin IX
IX
Nematostella vectensis Myosin III
Anopheles sinensis Myosin III
Anopheles darlingi Myosin III
III
Drosophila melanogaster Myosin V
Anopheles darlingi Myosin V
Xenopus laevis Myosin V
Nematostella vectensis Myosin V
V
Nematostella vectensis Myosin VI
Azumapecten farreri Myosin VI
Crassostrea gigas Myosin VI
VI
Nematostella vectensis MHC
Drosophila melanogaster MHC
Mus musculus MHC
Onchidium struma MHC
Dreissena rostriformis MHC
Mytilus galloprovincialis MHC
Argopecten irradians MHC
Placopecten magellanicus MHC
Acanthochitona fascicularis MHC
Octopus bimaculoides MHC
Todarodes pacificus MHC
Lingula anatina MHC
Platynereis dumerilii MHC
MHC
Pomacea canaliculata Myosin XVIII
Crassostrea virginica Myosin XVIII
Crassostrea gigas Myosin XVIII
XVIII
Nematostella vectensis Myosin I
Rattus norvegicus Myosin I
Anopheles darlingi Myosin I
I
100 76 100 42 96 100 100 47 100 100 100 28 97 100 100 100 100 97 100 100 82 57 75 47 76 82 100 55 100 57 95 100 56 100 100 100 100 100
0.4
B
520 530 540 550 560 570 580 590 600
Drosophila melanogaster MHC WTFIDFGMDLQLCIDLIEKPMGILSILEEESMFPKATDQTFSEKLTNTHLGKSAPFQKP-KPPKPGQQAAHFAIAHYAGCV
Nematostella vectensis MHC WEFIDFGHDLEPTINLI--
Platynereis dumerilii MHC WEFIDFGMDLQQCIDLIEKPMGILSILEEECMFPKASDDSFKAKMYENHLGKSPNFGKP-RPNKNAKFEAHFELKHYAGTV
Lingula anatina MHC WQFIDFGMDLQACIDLIEKPMGILSILEEECMFPKASDDTFKDKLYNNHMGKSPNFGKP-KPSKDRKHEAHFELYHYAGAV
Argopecten irradians MHC WEFIDFGMDLQMCIDLIEKPMGILSILEEECMFPKADDKSFQDKLYQNHMGKNRMFTKPGKPTRPNQGPAHFELHHYAGNV
Placopecten magellanicus MHC WEFIDFGMDLQMCIDLIEKPMGILSILEEECMFPKADDKSFQDYSYQNHIGKNRMFTKPGKPTRPNQGHAHFELHHYAGNV
Octopus bimaculoides MHC WEFIDFGLDLQACIDLIEKPMGILSILEEECMFPKASDTTFKNKLYDNHLGKNPMFGKP-KPPKPGQMEAHFALHHYAGSV
Todarodes pacificus MHC WEFIDFGLDLQACIELIEKPMGILSILEEECMFPKASDLSFKNKLYDNHLGKNPMFGKP-KPPKAGCIEAHFALHHYAGSV
Mytilus galloprovincialis MHC WEFIDFGMDLQACIDLIEKPMGILSILEEECMFPKADDKSFKDKLFANHLGKSPNFGRPGNASK-GKGQSDFELHHYAGIV
Dreissena rostriformis MHC WEFIDFGMDLQATIDLIEKPMGILSILEEECMFPKASDKTFLEKLYTNHLGKSNNFQKPTRSRK-GGADAHFELGHYAGAV
Acanthochitona fascicularis MHC WTFIDFGLDLQACIDLIEKPMGILSILEEECMFPKASDLTFKNKLYDNHMGKSPNFGKP-KPQK-GKAEAHFELHHYAGSV
Onchidium struma MHC WEFIDFGMDLQAAIDLIEKPMGILSILEEECMFPKASDKTFLDKLNGNHLGKSKNYGKAGKPKKAGQVEAHFELHHYAGSV

Suppl. Fig. S2

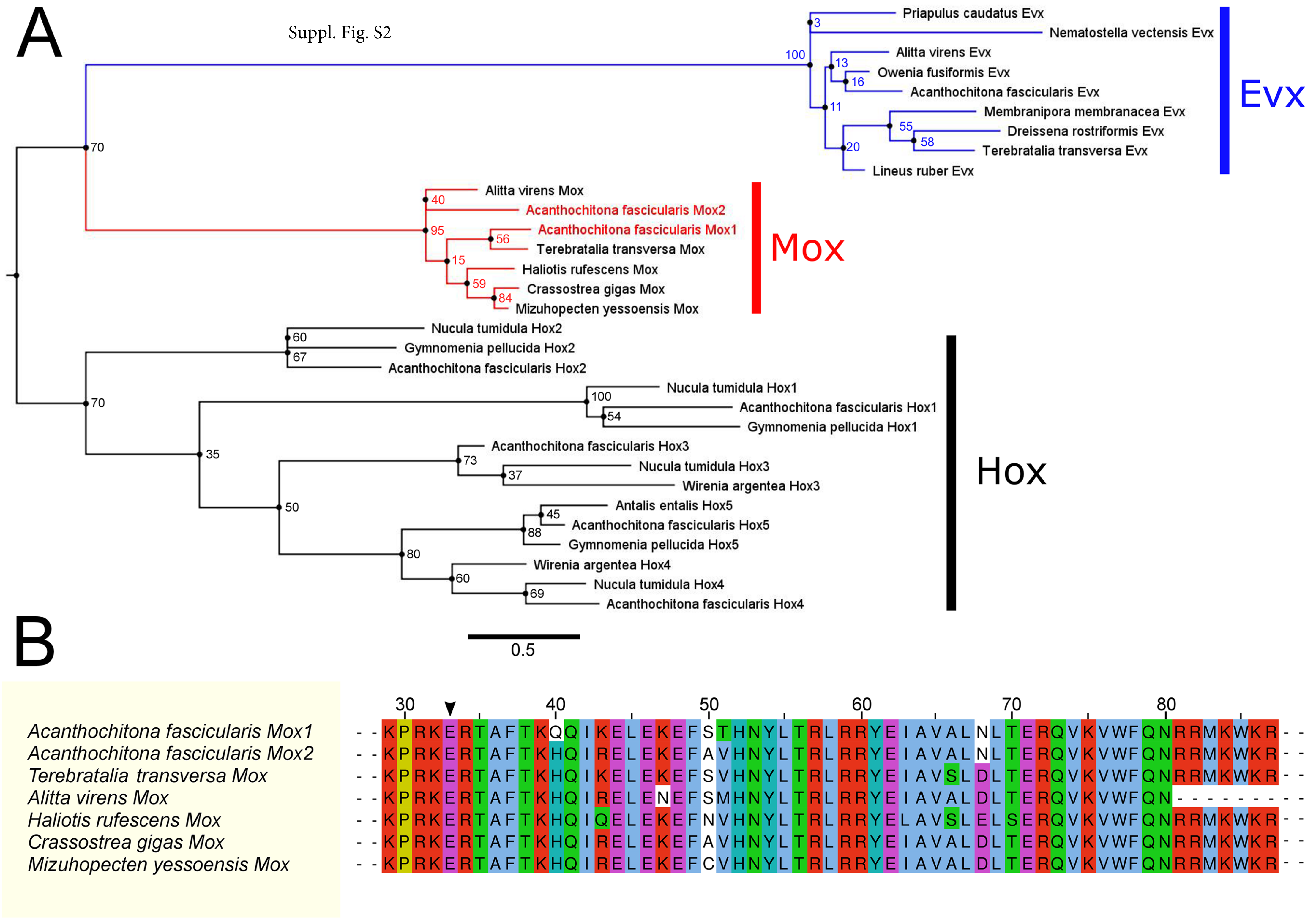
A
Priapulus caudatus Evx
Nematostella vectensis Evx
Alitta virens Evx
Owenia fusiformis Evx
Acanthochitona fascicularis Evx
Membranipora membranacea Evx
Dreissena rostriformis Evx
Terebratalia transversa Evx
Lineus ruber Evx
Evx
Alitta virens Mox
Acanthochitona fascicularis Mox2
Acanthochitona fascicularis Mox1
Terebratalia transversa Mox
Haliotis rufescens Mox
Crassostrea gigas Mox
Mizuhopecten yessoensis Mox
Mox
Nucula tumidula Hox2
Gymnomenia pellucida Hox2
Acanthochitona fascicularis Hox2
Nucula tumidula Hox1
Acanthochitona fascicularis Hox1
Gymnomenia pellucida Hox1
Acanthochitona fascicularis Hox3
Nucula tumidula Hox3
Wirenia argentea Hox3
Antalis entalis Hox5
Acanthochitona fascicularis Hox5
Gymnomenia pellucida Hox5
Wirenia argentea Hox4
Nucula tumidula Hox4
Acanthochitona fascicularis Hox4
Hox
0.5
B
30 40 50 60 70 80
Acanthochitona fascicularis Mox1 --KPRKERTAFTKQQIKELEKEFSTHNYLTRLRRYEIAVALNLTERQVKVWFQNRRMKWKR--
Acanthochitona fascicularis Mox2 --KPRKERTAFTKHQIRELEKEFAVHNYLTRLRRYEIAVALNLTERQVKVWFQNRRMKWKR--
Terebratalia transversa Mox --KPRKERTAFTKHQIKELEKEFSVHNYLTRLRRYEIAVSLDLTERQVKVWFQNRRMKWKR--
Alitta virens Mox --KPRKERTAFTKHQIRELENEFSMHNYLTRLRRYEIAVALDLTERQVKVWFQN----------
Haliotis rufescens Mox --KPRKERTAFTKHQIQELEKEFNVHNYLTRLRRYELAVSLELSERQVKVWFQNRRMKWKR--
Crassostrea gigas Mox --KPRKERTAFTKHQIRELEKEFAVHNYLTRLRRYEIAVALDLTERQVKVWFQNRRMKWKR--
Mizuhopecten yessoensis Mox --KPRKERTAFTKHQIRELEKEFCVHNYLTRLRRYEIAVALDLTERQVKVWFQNRRMKWKR--

Suppl. Fig. S3

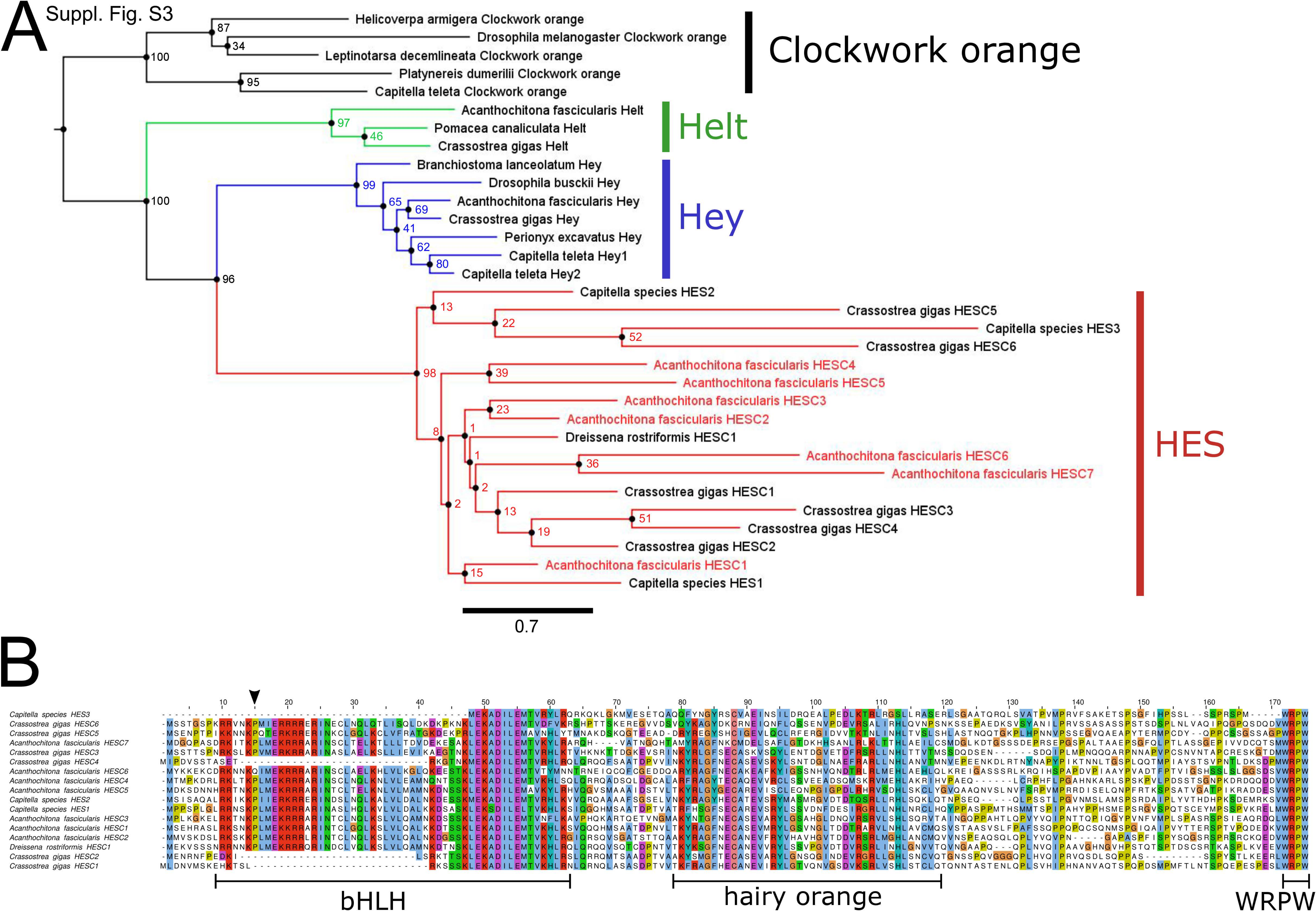

A
Helicoverpa armigera Clockwork orange
Drosophila melanogaster Clockwork orange
Leptinotarsa decemlineata Clockwork orange
Platynereis dumerilii Clockwork orange
Capitella teleta Clockwork orange
Acanthochitona fascicularis Helt
Pomacea canaliculata Helt
Crassostrea gigas Helt
Branchiostoma lanceolatum Hey
Drosophila busckii Hey
Acanthochitona fascicularis Hey
Crassostrea gigas Hey
Perionyx excavatus Hey
Capitella teleta Hey1
Capitella teleta Hey2
Capitella species HES2
Crassostrea gigas HESC5
Capitella species HES3
Crassostrea gigas HESC6
Acanthochitona fascicularis HESC4
Acanthochitona fascicularis HESC5
Acanthochitona fascicularis HESC3
Acanthochitona fascicularis HESC2
Dreissena rostriformis HESC1
Acanthochitona fascicularis HESC6
Acanthochitona fascicularis HESC7
Crassostrea gigas HESC1
Crassostrea gigas HESC3
Crassostrea gigas HESC4
Crassostrea gigas HESC2
Acanthochitona fascicularis HESC1
Capitella species HES1
Clockwork orange
Helt
Hey
HES
0.7
B
Capitella species HES3
Crassostrea gigas HESC6
Crassostrea gigas HESC5
Acanthochitona fascicularis HESC7
Crassostrea gigas HESC3
Crassostrea gigas HESC4
Acanthochitona fascicularis HESC6
Acanthochitona fascicularis HESC4
Acanthochitona fascicularis HESC5
Capitella species HES2
Capitella species HES1
Acanthochitona fascicularis HESC3
Acanthochitona fascicularis HESC1
Acanthochitona fascicularis HESC2
Dreissena rostriformis HESC1
Crassostrea gigas HESC2
Crassostrea gigas HESC1
bHLH
hairy orange
WRPW

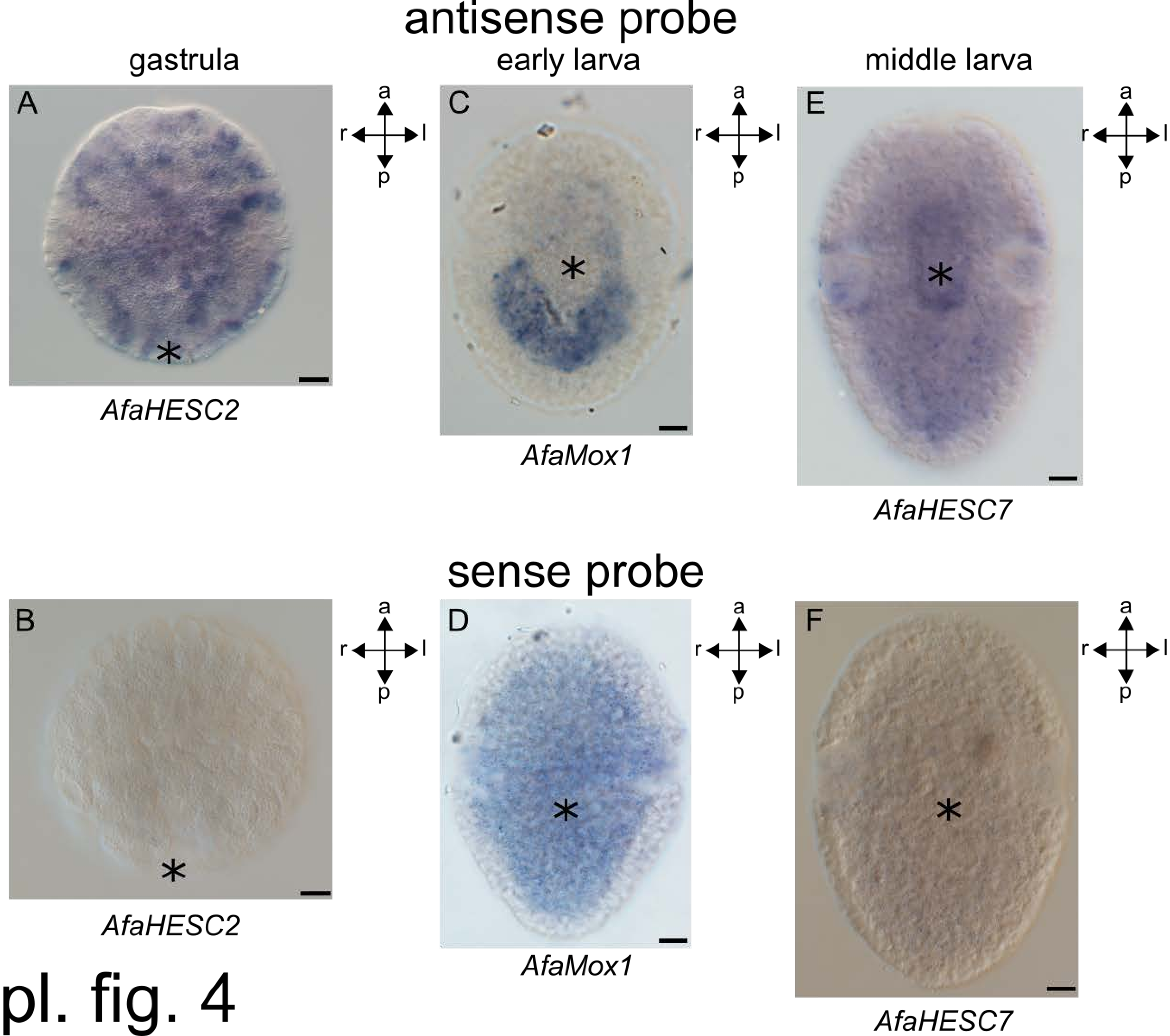
antisense probe
gastrula
early larva
middle larva
A
C
E
AfaHESC2
AfaMox1
AfaHESC7
sense probe
B
D
F
AfaHESC2
AfaMox1
AfaHESC7
a
p
r
l

suppl. fig. 4

Supplementary Table 1. Used primes for the gene amplification

Acanthochitona fascicularis primers		
Gene	Nucleotide sequence	
	F Primers	R Primers
AfaMHC	CTACGACAGGATGTTCAAG	CTTCGTCTGCTTCTCTTG
AfaMox1	GTTACATCACTATCCGCAC	CTCTGAGAAACTGTCATTTGG
AfaHESC1	CGAAGCTTCATCGAAATC	GTCTGTAATGAAGCCAGG
AfaHESC2	GATCTGTGGATATGGTCTC	CTTGGCTCTTCTGAGTTTG
AfaHESC3	CGATCACAGCCATTGAAG	GGTGTAGCACTGATTGATG
AfaHESC4	GAGGTTCACTCCACAATG	CTTGTTGATCACGGTCATC
AfaHESC5	CCATAGACGGACAAACAAG	CAGCTTGCTCTTCAGTTC
AfaHESC6	GGAGCACCTACAAGAAAG	CTCCACACAGAGTCAGAC
AfaHESC7	GGATAAATCGGAGGGTCC	CCTCCACATTGACGTTTG

Supplementary Table 2. Species list and GenBank accession numbers used for the *Myosin heavy chain* phylogeny.

Gene	Phylum	Species	Transcriptome database	NCBI accession	Original gene ID	New gene ID
Myosin II/heavy chain	Annelida	*Platynereis dumerilii*	-	AIJ28480.1	myosin heavy chain, partial	*Platynereis dumerilii MHC*
Myosin II/heavy chain	Arthropoda	*Drosophila melanogaster*	-	NP_523587.4	myosin heavy chain, isoform H	*Drosophila melanogaster MHC*
Myosin II/heavy chain	Brachiopoda	*Lingula anatina*	-	XP_023932278.1	myosin heavy chain, striated muscle isoform X26	*Lingula anatina MHC*
Myosin II/heavy chain	Cnidaria	*Nematostella vectensis*	[4, 5]	XP_001636958	myosin heavy chain, striated muscle isoform X1	*Nematostella vectensis MHC*
Myosin II/heavy chain	Craniata	*Mus musculus*	-	NP_001159682.1	myosin-15	*Mus musculus MHC*
Myosin II/heavy chain	Mollusca	*Acanthochitona fascicularis*	[6]	-	acr_tr62264_cds2_fwd	*Acanthochitona fascicularis MHC*
Myosin II/heavy chain	Mollusca	*Argopecten irradians*	-	AAC46490.1	myosin heavy chain	*Argopecten irradians MHC*
Myosin II/heavy chain	Mollusca	*Dreissena rostriformis*	[7]	GHRL01011381.1	Gene.143954	*Dreissena rostriformis MHC*
Myosin II/heavy chain	Mollusca	*Mytilus galloprovincialis*	-	CAB64662.1	myosin heavy chain, partial	*Mytilus galloprovincialis MHC*
Myosin II/heavy chain	Mollusca	*Octopus bimaculoides*	-	CDG41623.1	myosin heavy chain isoform A	*Octopus bimaculoides MHC*
Myosin II/heavy chain	Mollusca	*Onchidium struma*	-	AOR06339.1	myosin heavy chain	*Onchidium struma MHC*
Myosin II/heavy chain	Mollusca	*Placopecten magellanicus*	-	AAB03661.1	myosin heavy chain	*Placopecten magellanicus MHC*
Myosin II/heavy chain	Mollusca	*Todarodes pacificus*	-	ADU19853.1	myosin heavy chain	*Todarodes pacificus MHC*
Myosin I	Arthropoda	*Anopheles darlingi*	-	ETN59929.1	myosin i	*Anopheles darlingi Myosin I*

Myosin I	Cnidaria	*Nematostella vectensis*	[4, 5]	EDO35007.1	predicted protein, partial	*Nematostella vectensis Myosin I*
Myosin I	Craniata	*Rattus norvegicus*	-	CAA50871.1	myosin I	*Rattus norvegicus Myosin I*
Myosin III	Arthropoda	*Anopheles sinensis*	-	KFB46475.1	myosin iii	*Anopheles sinensis Myosin III*
Myosin III	Arthropoda	*Anopheles darlingi*	-	ETN67236.1	myosin III	*Anopheles darlingi Myosin III*
Myosin III	Cnidaria	*Nematostella vectensis*	[4, 5]	EDO45746	predicted protein	*Nematostella vectensis Myosin III*
Myosin V	Arthropoda	*Anopheles darlingi*	-	ETN63934.1	myosin v	*Anopheles darlingi Myosin V*
Myosin V	Arthropoda	*Drosophila melanogaster*	-	AAC99496.1	myosin V	*Drosophila melanogaster Myosin V*
Myosin V	Cnidaria	*Nematostella vectensis*	[4, 5]	EDO41819	predicted protein, partial	*Nematostella vectensis Myosin V*
Myosin VI	Cnidaria	*Nematostella vectensis*	[4, 5]	EDO36037	predicted protein	*Nematostella vectensis Myosin VI*
Myosin V	Craniata	*Xenopus laevis*	-	AFU81219.2	myosin V	*Xenopus laevis Myosin V*
Myosin VI	Mollusca	*Azumapecten farreri*	-	AAY63881.1	myosin VI, partial	*Azumapecten farreri VI*
Myosin VI	Mollusca	*Crassostrea gigas*	-	EKC25309.1	Myosin-VI	*Crassostrea gigas Myosin VI*
Myosin VII	Arthropoda	*Anopheles sinensis*	-	KFB45621.1	myosin vii	*Anopheles sinensis Myosin VII*
Myosin VII	Cnidaria	*Nematostella vectensis*	[4, 5]	EDO45563	predicted protein, partial	*Nematostella vectensis Myosin VII*
Myosin VII	Mollusca	*Crassostrea gigas*	-	EKC28928.1	Myosin-VIIa	*Crassostrea gigas Myosin VII*
Myosin VII	Mollusca	*Leptochiton asellus*	-	ASM47588.1	myosin VIIa	*Leptochiton asellus Myosin VII*
Myosin VII	Mollusca	*Pomacea canaliculata*	-	XP_025078688.1	myosin-VIIa-like isoform X1	*Pomacea canaliculata Myosin VII*
Myosin IX	Cnidaria	*Nematostella vectensis*	[4, 5]	EDO38834	predicted protein	*Nematostella vectensis Myosin IX*
Myosin IX	Craniata	*Mus musculus*	-	AAI38455.1	Myosin Ixb	*Mus musculus Myosin IX*
Myosin IX	Mollusca	*Crassostrea gigas*	-	XP_011417195.1	PREDICTED: unconventional myosin-IXa isoform X4	*Crassostrea gigas Myosin IX*
Myosin IX	Mollusca	*Pomacea canaliculata*	-	XP_025078508.1	unconventional myosin-IXa-like isoform X12	*Pomacea canaliculata Myosin IX*
Myosin XV	Arthropoda	*Anopheles sinensis*	-	KFB48001.1	myosin xv	*Anopheles sinensis Myosin XV*

Myosin XV	Mollusca	*Crassostrea gigas*	-	EKC41639.1	Myosin-XV	*Crassostrea gigas Myosin XV*
Myosin XV	Mollusca	*Crassostrea virginica*	-	XP_022313584.1	unconventional myosin-XV-like isoform X12	*Crassostrea virginica Myosin XV*
Myosin XVIII	Mollusca	*Crassostrea gigas*	-	XP_011450865.1	PREDICTED: unconventional myosin-XVIIIa isoform X1	*Crassostrea gigas Myosin XVIII*
Myosin XVIII	Mollusca	*Crassostrea virginica*	-	XP_022343135.1	unconventional myosin-XVIIIa-like isoform X1	*Crassostrea virginica Myosin XVIII*
Myosin XVIII	Mollusca	*Pomacea canaliculata*	-	XP_025090257.1	unconventional myosin-XVIIIa-like isoform X1	*Pomacea canaliculata Myosin XVIII*

Supplementary Table 3. Species list and GenBank accession numbers used for the *Mox* phylogeny.

Gene	Phylum	Species	Transcriptome database	NCBI accession	Original gene ID	New gene ID
Evx	Annelida	*Alitta virens*	-	AOS87315.1	even-skipped	*Alitta virens Evx*
Evx	Annelida	*Lineus ruber*	-	AMR72025.1	evx	*Lineus ruber Evx*
Evx	Annelida	*Owenia fusiformis*	-	AMY99557.1	evx	*Owenia fusiformis Evx*
Evx	Brachiopoda	*Terebratalia transversa*	-	AHY88463.1	evx	*Terebratalia transversa Evx*
Evx	Bryozoa	*Membranipora membranacea*	-	ARJ36942.1	even-skipped	*Membranipora membranacea Evx*
Evx	Cnidaria	*Nematostella vectensis*	-	SJX71987.1	Homeobox gene Evx, partial	*Nematostella vectensis Evx*
Evx	Mollusca	*Acanthochitona fascicularis*	[6]	-	acr_tr120485_cds1_fwd	*Acanthochitona fascicularis Evx*
Evx	Mollusca	*Dreissena rostriformis*	[7]	GHRL01002417.1	Gene.109262	*Dreissena rostriformis Evx*
Evx	Priapulida	*Priapulus caudatus*	-	AKU77019.1	evx	*Priapulus caudatus Evx*
Mox	Annelida	*Alitta virens*	-	AOS87316.1	mox, partial	*Alitta virens Mox*
Mox	Brachiopoda	*Terebratalia transversa*	-	AJV21315.1	mesoderm homeobox	*Terebratalia transversa Mox*
Mox	Mollusca	*Acanthochitona fascicularis*	[6]	-	acr_tr238840_cds2_rev	*Acanthochitona fascicularis Mox1*
Mox	Mollusca	*Acanthochitona fascicularis*	[6]	-	acr_tr159909_cds1_fwd	*Acanthochitona fascicularis Mox2*
Mox	Mollusca	*Crassostrea gigas*	-	EKC25209	Homeobox protein MOX-2	*Crassostrea gigas Mox*
Mox	Mollusca	*Haliotis rufescens*	-	CAA53027.1	Hrox1	*Haliotis rufescens Mox*
Mox	Mollusca	*Mizuhopecten yessoensis*	-	KP79_PYT03723	Homeobox protein MOX-2	*Mizuhopecten yessoensis Mox*
Hox1	Mollusca	*Acanthochitona fascicularis*	-	APD15641.1	homeobox hox 1	*Acanthochitona fascicularis Hox1*
Hox1	Mollusca	*Gymnomenia pellucida*	-	APD15663.1	homeobox hox 1	*Gymnomenia pellucida Hox1*
Hox1	Mollusca	*Nucula tumidula*	-	APD15698.1	homeobox hox 1	*Nucula tumidula Hox1*
Hox2	Mollusca	*Acanthochitona fascicularis*	-	APD15642.1	homeobox hox 2	*Acanthochitona fascicularis Hox2*
Hox2	Mollusca	*Gymnomenia pellucida*	-	APD15664.1	homeobox hox 2	*Gymnomenia pellucida Hox2*
Hox2	Mollusca	*Nucula tumidula*	-	APD15699.1	homeobox hox 2	*Nucula tumidula Hox2*
Hox3	Mollusca	*Acanthochitona fascicularis*	-	APD15643.1	homeobox hox 3	*Acanthochitona fascicularis Hox3*
Hox3	Mollusca	*Nucula tumidula*	-	APD15700.1	homeobox hox 3	*Nucula tumidula Hox3*
Hox3	Mollusca	*Wirenia argentea*	-	APD15711.1	homeobox hox 3	*Wirenia argentea Hox3*
Hox4	Mollusca	*Acanthochitona fascicularis*	-	APD15644.1	homeobox hox 4	*Acanthochitona fascicularis Hox4*

Hox4	Mollusca	*Nucula tumidula*	-	APD15701.1	homeobox hox 4	*Nucula tumidula Hox4*
Hox4	Mollusca	*Wirenia argentea*	-	APD15712.1	homeobox hox 4	*Wirenia argentea Hox4*
Hox5	Mollusca	*Acanthochitona fascicularis*	-	APD15645.1	homeobox hox 5	*Acanthochitona fascicularis Hox5*
Hox5	Mollusca	*Antalis entalis*	-	APD15655.1	homeobox hox 5	*Antalis entalis Hox5*
Hox5	Mollusca	*Gymnomenia pellucida*	-	APD15667.1	homeobox hox 5	*Gymnomenia pellucida Hox5*

Supplementary Table 4. Species list and GenBank accession numbers used for the *Hairy and enhancer of split* phylogeny.

Gene	Phylum	Species	Transcriptome database	NCBI accession	Original gene ID	New gene ID
HES	Annelida	*Capitella sp.*	-	DQ384620	hairy protein mRNA, complete cds.	*Capitella species HES1*
HES	Annelida	*Capitella sp.*	-	EU706455.1	HES2 (hes2) mRNA, partial cds.	*Capitella species HES2*
HES	Annelida	*Capitella sp.*	-	EU706456.1	HES3 (hes3) mRNA, partial cds.	*Capitella species HES3*
HES	Mollusca	*Acanthochitona fascicularis*	[6]	-	acr_tr122850_cds1_fwd	*Acanthochitona fascicularis HESC1*
HES	Mollusca	*Acanthochitona fascicularis*	[6]	-	acr_tr237216_cds4_rev	*Acanthochitona fascicularis HESC2*
HES	Mollusca	*Acanthochitona fascicularis*	[6]	-	acr_tr288909_cds2_rev	*Acanthochitona fascicularis HESC3*
HES	Mollusca	*Acanthochitona fascicularis*	[6]	-	acr_tr146030_cds2_rev	*Acanthochitona fascicularis HESC4*
HES	Mollusca	*Acanthochitona fascicularis*	[6]	-	acr_tr264437_cds1_fwd	*Acanthochitona fascicularis HESC5*
HES	Mollusca	*Acanthochitona fascicularis*	[6]	-	acr_tr284209_cds1_fwd	*Acanthochitona fascicularis HESC6*
HES	Mollusca	*Acanthochitona fascicularis*	[6]	-	acr_tr239548_cds1_fwd	*Acanthochitona fascicularis HESC7*
HES	Mollusca	*Dreissena rostriformis*	[7]	GHRL01019498.1	Gene.33105	*Dreissena rostriformis HESC1*
HES	Mollusca	*Crassostrea gigas*	[8]	EKC30677	Transcription factor HES-1 [obsolete]	*Crassostrea gigas HESC1*
HES	Mollusca	*Crassostrea gigas*	[8]	EKC30678	Transcription factor HES-1-B [obsolete]	*Crassostrea gigas HESC2*
HES	Mollusca	*Crassostrea gigas*	[8]	EKC23398	Transcription factor HES-1-B [obsolete]	*Crassostrea gigas HESC3*
HES	Mollusca	*Crassostrea gigas*	[8]	EKC23396	Transcription factor HES-1 [obsolete]	*Crassostrea gigas HESC4*

HES	Mollusca	*Crassostrea gigas*	8	EKC43182	Transcription factor HES-1 [obsolete]	*Crassostrea gigas HESC5*
HES	Mollusca	*Crassostrea gigas*	8	EKC30676	Transcription factor HES-1 [obsolete]	*Crassostrea gigas HESC6*
Hey	Annelida	*Capitella teleta*	-	ELU08981	hypothetical protein CAPTEDRAFT_182542	*Capitella teleta Hey1*
Hey	Annelida	*Capitella teleta*	-	ELU08980	hypothetical protein CAPTEDRAFT_228069	*Capitella teleta Hey2*
Hey	Annelida	*Perionyx excavatus*	-	ASQ42633.1	HEY, partial	*Perionyx excavatus Hey*
Hey	Arthropoda	*Drosophila busckii*	-	ALC41030.1	Hey	*Drosophila busckii Hey*
Hey	Cephalochordata	*Branchiostoma lanceolatum*	-	AWV91612.1	hey	*Branchiostoma lanceolatum Hey*
Hey	Mollusca	*Acanthochitona fascicularis*	6	-	acr_tr163747_cds1_fwd	*Acanthochitona fascicularis Hey*
Hey	Mollusca	*Crassostrea gigas*	8	EKC34248	Hairy/enhancer-of-split related with YRPW motif protein 1 [obsolete]	*Crassostrea gigas Hey*
Helt	Mollusca	*Acanthochitona fascicularis*	6	-	acr_tr149761_cds1_fwd	*Acanthochitona fascicularis Helt*
Helt	Mollusca	*Crassostrea gigas*	8	EKC36048	Hairy and enhancer of split-related protein HELT [obsolete]	*Crassostrea gigas Helt*
Helt	Mollusca	*Pomacea canaliculata*	-	XP_025115546.1	HES-related protein helt-like	*Pomacea canaliculata Helt*
Clockwork orange	Annelida	*Capitella teleta*	-	ELU02556	hypothetical protein CAPTEDRAFT_196737	*Capitella teleta Clockwork orange*
Clockwork orange	Annelida	*Platynereis dumerilii*	-	AGS55449	hairy enhancer of split related	*Platynereis dumerilii Clockwork orange*
Clockwork orange	Arthropoda	*Drosophila melanogaster*	-	NP_001247025.1	clockwork orange, isoform C	*Drosophila melanogaster Clockwork orange*
Clockwork orange	Arthropoda	*Helicoverpa armigera*	-	ARQ15181.1	clockwork orange	*Helicoverpa armigera Clockwork orange*
Clockwork orange	Arthropoda	*Leptinotarsa decemlineata*	-	AKG92774.1	clockwork orange	*Leptinotarsa decemlineata Clockwork orange*

Supplementary Table 5. Number of investigated genes and their expression domains of the Mox, HES, and MHC families in metazoan phyla.

Gene family	Phylum	Species	Number of genes	Expression	Reference
Mox	Mollusca	*Acanthochitona fascicularis* (Polyplacophora)	2	mesodermal bands, ventrolateral muscle	present study
	Mollusca	*Haliotis asinina* (Gastropoda)	1	mesodermal bands, foot musculature	9
	Annelida	*Alitta virens*	1	mesodermal bands, precursor cells of musculature	10
	Brachiopoda	*Terebratalia transversa*	1	mesodermal bands	11
	Hexapoda	*Drosophila melanogaster*	1	dorsal median cells	12
	Nematoda	*Caenorhabditis elegans*	0	loss of Mox ortholog	13
	Echinodermata	*Strongylocentrotus purpuratus*	1	neurogenesis, apical organ	14
	Hemichordata	*Saccoglossus kowalevskii*	1	ventral mesoderm	15
	Chordata	*Branchiostoma floridae*	1	somitogenesis	16
	Chordata	*Mus musculus*	2	mesodermal compartments, organogenesis	17
	Plathyhelminthes	*Xenacoelomorpha*	?	not investigated	not investigated
	Cnidaria	*Nematostella vectensis*	4	anterior endoderm	18
HES	Mollusca	*Acanthochitona fascicularis* (Polyplacophora)	7	mesoderm, neurogenesis, gut formation	present study
	Mollusca	*Crepidula fornicata*	2	ectoderm, neurogenesis, gut formation	19
	Annelida	*Capitella teleta*	3	segmentation, chaetogenesis, neurogenesis, gut formation	20
	Annelida	*Platynereis dumerilii*	13		21
	Brachiopoda	*Terebratalia transversa*	2	mesoderm, ectoderm, chaetogenesis,	22
	Hexapoda	*Drosophila melanogaster*	1	segmentation	23-25
	Nematoda	*Caenorhabditis elegans*	1	neurogenesis	26
	Echinodermata	*Strongylocentrotus purpuratus*	1	ectoderm	27
	Chordata	*Branchiostoma belcheri*	8	somitogenesis, neurogenesis, gut formation	28
	Chordata	*Mus musculus*	6		29
	Chordata	*Xenopus laevis*	3	induction and migration of neural crest	30
	Plathyhelminthes	*Symsagittifera roscoffensis*	1	neurogenesis	31
	Cnidaria	*Nematostella vectensis*	4	ectoderm, endoderm	32
	Cnidaria	*Hydra vulgaris*	1	budding	33

MHC	Mollusca	*Acanthochitona fascicularis (Polyplacophora)*	1	musculature	present study
	Annelida	*Platynereis dumerilii*	1		34
	Planaria	*Schmidtea polychroa*	1		35
	Hexapoda	*Drosophila melanogaster*	1		36
	Nematoda	*Caenorhabditis elegans*	4		37,38
	Echinodermata	*Strongylocentrotus purpuratus*	1		39
	Chordata	*Branchiostoma belcheri*	2		40
	Chordata	*Danio rerio*	2		41
	Cnidaria	*Nematostella vectensis*	1		42

Supplementary references

1. Larsson, A. AliView: A fast and lightweight alignment viewer and editor for large datasets. *Bioinformatics* **30**, 3276–3278 (2014).

2. Waterhouse, A. M., Procter, J. B., Martin, D. M. A., Clamp, M. & Barton, G. J. Jalview Version 2-a multiple sequence alignment editor and analysis workbench. *Bioinformatics* **25**, 1189–1191 (2009).

3. Rambaut, A. FigTree 1.4. 2 software. *Inst. Evol. Biol. Univ. Edinburgh* http://tree.bio.ed.ac.uk/software/figtree/ (2014).

4. Putnam, N. H. et al. Sea anemone genome reveals ancestral eumetazoan gene repertoire and genomic organization. *Science* **317**, 86–94 (2007).

5. Sullivan, J. C., Reitzel, A. M. & Finnerty, J. R. Upgrades to StellaBase facilitate medical and genetic studies on the starlet sea anemone, *Nematostella vectensis*. *Nucleic Acids Res*. **36**, D607–D611 (2008).

6. De Oliveira, A. L. et al. Comparative transcriptomics enlarges the toolkit of known developmental genes in mollusks. *BMC Genom*. **17**, 1–23 (2016).

7. Calcino, A. D. et al. The quagga mussel genome and the evolution of freshwater tolerance. *DNA Res.* **26**, 411–422 (2019).

8. Wang, J. et al. The oyster genome reveals stress adaptation and complexity of shell formation. *Nature* **490**, 49–54 (2012).

9. Hinman, V. F. & Degnan, B. M. Mox homeobox expression in muscle lineage of the gastropod *Haliotis asinina*: Evidence for a conserved role in bilaterian myogenesis. *Dev. Genes Evol*. **212**, 141–144 (2002).

10. Kozin, V. V., Filimonova, D. A., Kupriashova, E. E. & Kostyuchenko, R. P. Mesoderm patterning and morphogenesis in the polychaete *Alitta virens* (Spiralia, Annelida): Expression of mesodermal markers Twist, Mox, Evx and functional role for MAP kinase signaling. *Mech. Dev*. **140**, 1–11 (2016).

11. Passamaneck, Y. J., Hejnol, A. & Martindale, M. Q. Mesodermal gene expression during the embryonic and larval development of the articulate brachiopod *Terebratalia transversa*. *EvoDevo* **6**, 1–21 (2015).

12. Chiang, C., Patel, N.H., Young, K.E. & Beachy, P.A. The novel homeodomain gene *buttonless* specifies differentiation and axonal guidance functions of *Drosophila* dorsal median cells. *Development* **120**, 3581–93 (1994).

13. Ruvkun, G. et al. The Taxonomy of Developmental Control in *Caenorhabditis elegans*. *Science* **282**, 2033–2041 (1998).

14. Poustka, A.J. et al. A global view of gene expression in lithium and zinc treated sea urchin embryos: New components of gene regulatory networks. *Genome Biol*. **8**, R85 (2007).

15. Lowe, C.J. et al. Dorsoventral patterning in hemichordates: Insights into early chordate evolution. *PLoS Biol*. **4**, 1603–1619 (2006).

16. Minguillón, C. & Garcia-Fernàndez, J. The single amphioxus Mox gene: Insights into the functional evolution of Mox genes, somites, and the asymmetry of amphioxus somitogenesis. *Dev. Biol*. **246**, 455–465 (2002).

17. Candia, A.F. et al. Mox-1 and Mox-2 define a novel homeobox gene subfamily and are differentially expressed during early mesodermal patterning in mouse embryos. *Development* **116**, 1123–36 (1992).

18. Ryan, J.F. et al. The cnidarian-bilaterian ancestor possessed at least 56 homeoboxes: Evidence from the starlet sea anemone, *Nematostella vectensis*. *Genome Biol*. **7**, R64 (2006).

19. Perry, K.J. et al. Deployment of regulatory genes during gastrulation and germ layer specification in a model spiralian mollusc *Crepidula*. *Dev. Dyn*. **244**, 1215–1248 (2015).

20. Thamm, K. & Seaver, E.C. Notch signaling during larval and juvenile development in the polychaete annelid *Capitella sp*. I. *Dev. Biol.* **320**, 304–318 (2008).

21. Gazave, E., Guillou, A. & Balavoine, G. History of a prolific family: The Hes/Hey-related genes of the annelid *Platynereis*. *Evodevo* **5**, 1–33 (2014).

22. Schiemann, S.M. et al. Clustered brachiopod Hox genes are not expressed collinearly and are associated with lophotrochozoan novelties. *Proc. Natl. Acad. Sci*. **114**, E1913–E1922 (2017).

23. Lardelli, M. & Ish-Horowicz, D. Drosophila hairy pair-rule gene regulates embryonic patterning outside its apparent stripe domains. *Development* **118**, 255–266 (1993).

24. Frise, E., Hammonds, A.S. & Celniker, S.E. Systematic image-driven analytics of the spatial *Drosophila* embryonic expression landscape. *Mol. Syst. Biol*. **6**, 345 (2010).

25. Zhan, Y., Maung, S.W., Shao, B. & Myat, M.M. The bHLH transcription factor, Hairy, refines the terminal cell fate in the *Drosophila* embryonic trachea. *PLoS One* **5**, e14134 (2010).

26. Lanjuin, A., Claggett, J., Shibuya, M., Hunter, C. & Sengupta, P. Regulation of neuronal lineage decisions by the HES-related bHLH protein REF-1. *Dev. Biol.* **290**, 139-151 (2006).

27. Carroll, S.B., Laughon, A. & Thalley, B.S. Expression, function, and regulation of the hairy segmentation protein in the *Drosophila* embryo. *Genes Dev*. **2**, 883–890 (1988).

28. Jiménez-Delgado, S., Crespo, M., Permanyer, J., Garcia-Fernàndez, J. & Manzanares, M. Evolutionary genomics of the recently duplicated amphioxus Hairy genes. *Int. J. Biol. Sci*. **2**, 66–72 (2006).

29. Kageyama, R., Ohtsuka, T. & Kobayashi, T. The Hes gene family: repressors and oscillators that orchestrate embryogenesis. *Development* **134**, 1243–1251 (2007).

30. Vega-López et al. Functional analysis of Hairy genes in Xenopus neural crest initial specification and cell migration. *Dev. Dyn.* **244**, 988–1013 (2015).

31. Perea-Atienza, E., Sprecher, S.G. & Martínez, P. Characterization of the bHLH family of transcriptional regulators in the acoel *S. roscoffensis* and their putative role in neurogenesis. *EvoDevo* **9**, 1-16 (2018).

32. Marlow, H., Roettinger, E., Boekhout, M. & Martindale, M.Q. Functional roles of Notch signaling in the cnidarian *Nematostella vectensis*. *Dev. Biol.* **362**, 295–308 (2012).

33. Münder, S. et al. Notch signalling defines critical boundary during budding in Hydra. *Dev. Biol.* **344**, 331–345 (2010).

34. Pfeifer, K., Schaub, C., Domsch, K., Dorresteijn, A. & Wolfstetter, G. Maternal inheritance of Twist and Analysis of MAPK activation in embryos of the polychaete annelid *Platynereis dumerilii*. *PLoS One* **9**, 1–9 (2014).

35. Cardona, A., Fernández, J., Solana, J. & Romero, R. An in situ hybridization protocol for planarian embryos: Monitoring myosin heavy chain gene expression. *Dev. Genes Evol.* **215**, 482–488 (2005a).

36. Zhang, S. & Bernstein, S.I. Spatially and temporally regulated expression of myosin heavy chain alternative exons during Drosophila embryogenesis. *Mech. Dev.* **101**, 35–45 (2001).

37. Mackenzie, J.M., Schachat, F. & Epstein, H.F. Immunocytochemical localization of two myosins within the same muscle cells in *Caenorhabditis elegans*. *Cell* **15**, 413–419 (1978).

38. Bejsovec, A. & Anderson, P. Myosin heavy-chain mutations that disrupt *Caenorhabditis elegans* thick filament assembly. *Genes Dev.* **2**, 1307–1317 (1988).

39. Andrikou, C., Pai, C.Y., Su, Y.H. & Arnone, M.I. Logics and properties of a genetic regulatory program that drives embryonic muscle development in an echinoderm. *Elife* **4**, 1–22 (2015).

40. Urano, A., Suzuki, M.M., Zhang, P., Satoh, N. & Satoh, G. Expression of muscle-related genes and two MyoD genes during amphioxus notochord development. *Evol. Dev.* **5**, 447–458 (2003).

41. McGuigan, K., Phillips, P.C. & Postlethwait, J.H. Evolution of sarcomeric myosin heavy chain genes: Evidence from fish. *Mol. Biol. Evol.* **21**, 1042–1056 (2004).

42. Renfer, E., Amon-Hassenzahl, A., Steinmetz, P.R.H. & Technau, U. A muscle-specific transgenic reporter line of the sea anemone, *Nematostella vectensis*. *Proc. Natl. Acad. Sci.* **107**, 104–108 (2010).
